# Discovery of novel disease-causing mutation in *SSBP1* and its correction using adenine base editor to improve mitochondrial function

**DOI:** 10.1016/j.omtn.2024.102257

**Published:** 2024-06-17

**Authors:** Ju Hyuen Cha, Seok-Hoon Lee, Yejin Yun, Won Hoon Choi, Hansol Koo, Sung Ho Jung, Ho Byung Chae, Dae Hee Lee, Seok Jae Lee, Dong Hyun Jo, Jeong Hun Kim, Jae-Jin Song, Jong-Hee Chae, Jun Ho Lee, Jiho Park, Jin Young Kang, Sangsu Bae, Sang-Yeon Lee

**Affiliations:** 1 Department of Otorhinolaryngology, Seoul National University College of Medicine, Seoul National University Hospital, Seoul, Republic of Korea; 2 Department of Biomedical Sciences, Seoul National University College of Medicine, Seoul, Republic of Korea; 3 Cancer Research Institute, Seoul National University College of Medicine, Seoul, Republic of Korea; 4 CTCELLS, Inc, Daejeon, Republic of Korea; 5 Fight Against Angiogenesis-Related Blindness (FARB) Laboratory, Clinical Research Institute, Seoul National University Hospital, Seoul, Republic of Korea; 6 Department of Ophthalmology, Seoul National University College of Medicine, Seoul, Republic of Korea; 7 Department of Anatomy and Cell Biology, Seoul National University College of Medicine, Seoul, Republic of Korea; 8 Department of Otorhinolaryngology, Seoul National University College of Medicine, Seoul National University Bundang Hospital, Seongnam, Republic of Korea; 9 Department of Genomic Medicine, Seoul National University Hospital, Seoul, Republic of Korea; 10 Department of Chemistry, Korea Advanced Institute of Science and Technology, Daejeon 34141, Republic of Korea; 11 Medical Research Center of Genomic Medicine Institute, Seoul National University College of Medicine, Seoul, Republic of Korea; 12 Sensory Organ Research Institute, Seoul National University Medical Research Center, Seoul, Republic of Korea

**Keywords:** MT: RNA/ DNA editing, mitochondrial diseases, sensorineural deafness, optic atrophy, myopathy, single-stranded binding protein 1, SSBP1, NG-Cas9-based ABE8e, NG-ABE8e, NG-Cas9-based ABE8eWQ, NG-ABE8eWQ, editing efficacy, off-target effects

## Abstract

Mutations in nuclear genes regulating mitochondrial DNA (mtDNA) replication are associated with mtDNA depletion syndromes. Using whole-genome sequencing, we identified a heterozygous mutation (c.272G>A:p.Arg91Gln) in single-stranded DNA-binding protein 1 (SSBP1), a crucial protein involved in mtDNA replisome. The proband manifested symptoms including sensorineural deafness, congenital cataract, optic atrophy, macular dystrophy, and myopathy. This mutation impeded multimer formation and DNA-binding affinity, leading to reduced efficiency of mtDNA replication, altered mitochondria dynamics, and compromised mitochondrial function. To correct this mutation, we tested two adenine base editor (ABE) variants on patient-derived fibroblasts. One variant, NG-Cas9-based ABE8e (NG-ABE8e), showed higher editing efficacy (≤30%) and enhanced mitochondrial replication and function, despite off-target editing frequencies; however, risks from bystander editing were limited due to silent mutations and off-target sites in non-translated regions. The other variant, NG-Cas9-based ABE8eWQ (NG-ABE8eWQ), had a safer therapeutic profile with very few off-target effects, but this came at the cost of lower editing efficacy (≤10% editing). Despite this, NG-ABE8eWQ-edited cells still restored replication and improved mtDNA copy number, which in turn recovery of compromised mitochondrial function. Taken together, base editing-based gene therapies may be a promising treatment for mitochondrial diseases, including those associated with *SSBP1* mutations.

## Graphical abstract

**Figure:**
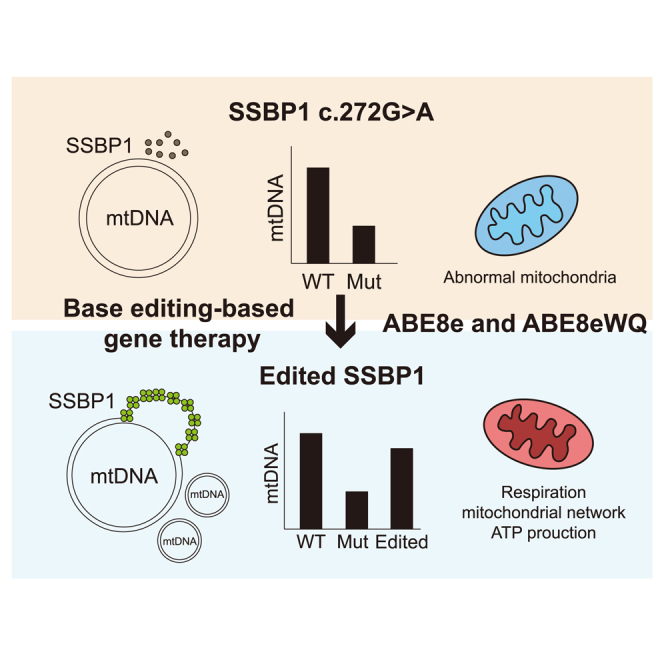

## Teaser

Base editing, capable of inducing precise base transitions with high efficacy, has evolved to enhance precision in treating diseases. This study corrected a disease-causing *SSBP1* mutation, essential for mitochondrial replication, using a specialized base editor designed to minimize off-target effects, accelerating clinical applications for mitochondrial diseases.

## Introduction

Mitochondrial diseases are characterized by respiratory chain dysfunction and often manifest as multisystemic involvement, leading to a spectrum of symptoms.^1^ These conditions often result from mutations in either the mitochondrial DNA (mtDNA) or nuclear genomes.^2^ The mtDNA is a compact, double-stranded circular DNA with a high copy number. It encodes 37 essential genes, which include 13 core protein components of the mitochondrial respiratory chain complexes (complexes I–V), as well as 2 ribosomal RNAs and 22 transfer RNAs necessary for their translation. Each cell contains thousands of copies of mtDNA, and these are organized into nucleoids, each carrying approximately 1,000 molecules of the packaging protein and the replisome machinery.^3^ Notably, the nuclear genome encodes more than 99% of the mitochondrial proteome, encompassing not only respiratory chain subunits, but also all the proteins responsible for mtDNA maintenance, replication, transcription, and copy number control of the mitochondrial genome.^4^^,^^5^^,^^6^^,^^7^^,^^8^ Mutations in these nuclear genes can induce instability of the mitochondrial genome, as characterized by mtDNA depletion and somatic accumulation of multiple deletions in post-mitotic tissues, which in turn lead to mitochondrial diseases inherited in a Mendelian fashion.^9^ While our understanding of mitochondrial biology and pathology has significantly advanced, the complex nature of mitochondrial inheritance combined with the contributions of nuclear and mitochondrial genomes has made it challenging to fully elucidate the mechanisms underlying mitochondrial diseases.^10^

Human mtDNA is replicated by specialized machinery distinct from the nuclear replisome.^11^ In particular, the mitochondrial replisome, a complex multi-component apparatus, is pivotal for mtDNA replication.^12^ Mutations in mitochondrial replication machinery can lead to mtDNA depletion, which can sometimes lead to deletions within the mitochondria genome.^13^ One of components of the mtDNA replication complex, mitochondrial single-stranded DNA-binding protein 1 (SSBP1), is crucial for regulating mtDNA replication initiation and maintenance in mammalian mitochondria.^14^ In humans, pathogenic mutations in *SSBP1* have been found to impair mtDNA replication fidelity and cause mtDNA depletion and/or deletion in mitochondria genome, which subsequently led to compromised oxidative phosphorylation and a spectrum of phenotypes in patients.^15^^,^^16^ Until now, fewer than 15 mutations in *SSBP1* have been identified,^17^ and the majority of these patients present with optic atrophy, sensorineural deafness, mitochondrial myopathy, and kidney failure.

Despite advancements in our understanding of the diagnosis and pathology of mitochondrial diseases, a definitive treatment remains elusive. Gene therapy has produced clinical benefits for several human diseases, and gene-editing technologies are expected to play a major role in the field’s future.^18^ In particular, CRISPR-based technologies are currently being developed for mammalian genome editing to treat a number of genetic disorders.^19^ The canonical CRISPR nucleases generate DNA double-stranded breaks (DSBs) at target sites, which are then repaired mostly by non-homologous end-joining and homology-directed repair (HDR). While HDR can correct disease-causing mutations, CRISPR-mediated DSBs can have unintended outcomes, such as small insertions and/or deletions (indels), as well as large DNA deletions, chromosomal depletion, and p53-driven programmable cell death.^20^^,^^21^ Alternatively, base editors that consist of Cas9 nickase and cytidine or adenosine deaminases were developed, which generate single-strand breaks instead of DSBs.^20^^,^^22^^,^^23^^,^^24^^,^^25^ In general, cytosine base editors (CBEs) and adenine base editors (ABEs) can introduce C·G to T·A and A·T to G·C substitutions, respectively, with high efficiency. Given that point mutations represent more than one-half of all identified pathogenic genetic variants in humans,^26^ base editors possess significant therapeutic potential for correcting disease-causing mutations. At present, base editors are undergoing clinical trials for several rare diseases^27^; thus, mutations associated with mitochondrial diseases may be promising candidates for base editing-based gene therapy.

In this study, we identified a novel heterozygous mutation in *SSBP1* (c.272G>A:p.Arg91Gln) in one family exhibiting symptoms of sensorineural deafness, optic atrophy, macular dystrophy, early cataract, and mitochondrial myopathy, which are all compatible with mitochondria disease. We revealed that this *SSBP1* mutation altered multimer formation and reduced DNA-binding affinity, which compromised mtDNA replication fidelity and altered mitochondria dynamics. Ultimately, this *SSBP1* mutation led to mtDNA depletion and subsequent mitochondrial dysfunction. Our results replicate findings observed in *SSBP1* mutations. To rescue mitochondrial function, we tested two ABE variants in patient cells harboring the *SSBP1* mutation and assessed editing efficacies, off-target effects, and functional recovery. Taken together, our findings suggest base editing-based gene therapies may be beneficial for mitochondrial diseases and provide guidance for selecting appropriate base editors for potential clinical use.

## Results

### Identification of *SSBP1* mutation and associated clinical phenotype

**Figure 1:**
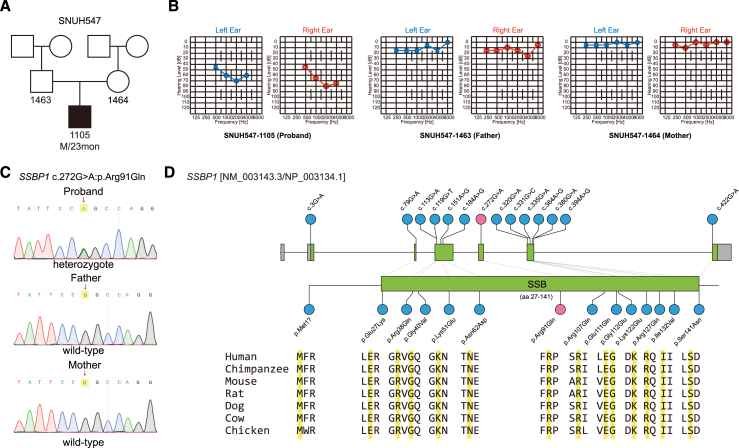
Characterization of *de novo* mutation in *SSBP1* (A) The pedigree of the Korean family (SNUH547) harboring a *de novo* heterozygous *SSBP1* mutation (c.272G>A:p.Arg91Gln). Filled symbols and opened symbols indicate affected and unaffected individuals, respectively. (B) The auditory steady-state response for the proband (SNUH547-1105) exhibits bilateral, symmetric, moderately severe sensorineural deafness. Meanwhile, pure-tone audiometry for both parents shows normal hearing. (C) Sanger sequencing chromatograms of the *SSBP1* c.272G>A:p.Arg91Gln mutation in the SNUH547 family. The arrow indicates the site of the mutation. (D) The sequence domain (top) and conservation maps (bottom) showcase *SSBP1* mutations previously reported in the literature (blue circles), with the inclusion of the novel mutation (c.272G>A:p.Arg91Gln) from this study (red circle). In the conservation map, gray regions denote untranslated regions, while green regions signify the coding sequence. All the mutations’ residues, including Arg91 residue, were highly conserved among the *SSBP1* orthologs in various species (highlighted in yellow).

The 2-year-old proband (SNUH547-1105) was a sporadic case in family SNUH547 (Figure 1A), exhibiting symmetrical moderate-to-severe, down-sloping sensorineural deafness, confirmed via electrophysiological auditory tests (Figure 1B). Both transient-evoked otoacoustic emissions and distortion product otoacoustic emissions were undetectable across all frequencies (Figure S3A). Over the following 2 years, hearing loss progressively worsened. Radiological imaging revealed no anomalies in the inner ear or brain. Additionally, tests for congenital cytomegalovirus infection yielded normal results. Meanwhile, the parent’s pure-tone audiograms were normal (Figure 1B). Using a stepwise genomic approach, we performed trio whole-genome sequencing (WGS) and identified a *de novo* heterozygous missense mutation (c.272G>A:p.Arg91Gln) in the *SSBP1* gene. Sanger sequencing in parental samples confirmed that the mutation arose *de novo* (Figure 1C), consistent with phenotypes observed in the family. We examined several population databases, including Genome Aggregation Database and the Korean Variant Archive, but could not find any reports of this missense mutation (p.Arg91Gln). The Arg91 residue is conserved across *SSBP1* orthologs of multiple species (http://genome.ucsc.edu/) (Figure 1D). *In silico* analyses also predicted this mutation was disease-causing, with a Combined Annotation Dependent Depletion phred score of 23.9. Based on the American College of Medical Genetics and Genomics/Association for Molecular Pathology (ACMG/AMP) guidelines,^28^ the *SSBP1* mutation (c.272G>A:p.Arg91Gln) is also considered pathogenic (Table S1).

In the literature, 10 studies have identified 13 distinct missense mutations in *SSBP1* (Figure 1D; Table S2), all of which are associated with mtDNA depletion syndrome. Sensorineural deafness was frequently reported (Figure S1), consistent with the localization of SSBP1 expression in the inner ear (Figure S2). Optic atrophy is the predominant phenotype observed in patients with *SSBP1* mutations, accompanied by a range of neurological symptoms. Upon slit-lamp examination and ultrasound examination on our proband (SNUH547-1105), remarkable lens opacity affecting the central visual axis was observed in both eyes (Figure S3B, left, top, and bottom). In the fundus examination, lens opacity resulted in a blurry appearance of the macula, which is an important anatomical area for central vision (Figure 1F, left and middle). The patient underwent bilateral lensectomy and anterior vitrectomy at 1-month intervals. At the 4–5 months post-operative follow-up examination, the Snellen visual acuity significantly improved to 20/32 in the right eye and 20/40 in the left eye. In parallel, slit lamp examination and ultrasound examination confirmed clear removal of lens opacity (Figure S3B, right, top and bottom). At this stage, fundus examination revealed optic disc atrophy and degeneration of the retinal pigment epithelium in the macula (Figure S3B, right and middle). Additional follow-up by neurologists identified mild muscle weakness, which was consistent with the detection of CD56-positive degenerated myocytes by muscle biopsy (Figure S3C). These results suggested a mild mitochondrial myopathy, although overt muscle phenotypes were not observed, and creatine kinase levels remained within the normal range. Overall, the patient exhibited signs of sensorineural deafness, congenital cataract, optic atrophy, macular dystrophy, and myopathy, all of which were indicative of mitochondrial disease.

In the literature, mtDNA depletion has been observed in the majority of patients with mitochondrial disease (Table S2), whereas mtDNA deletions have been specifically reported in cases with double hits (*SSBP1* c.3G>A and *MT-RNR1* m.1555A>G)^29^ and in a patient with an *SSBP1* c.79G>A mutation.^30^ In our patient (SNUH547-1105), neither mtDNA panel sequencing nor multiplex genomic ligation-dependent probe amplification of genomic DNA extracted from peripheral blood samples and patient-derived fibroblasts identified any pathogenic mtDNA variants or copy number variations in the mitochondria genome (Figure S4). Additionally, long-range PCR performed on skeletal muscle confirmed the absence of mtDNA deletions (Figure S5).

### Overexpression of mutant SSBP1 decreases mtDNA copy number

**Figure 2:**
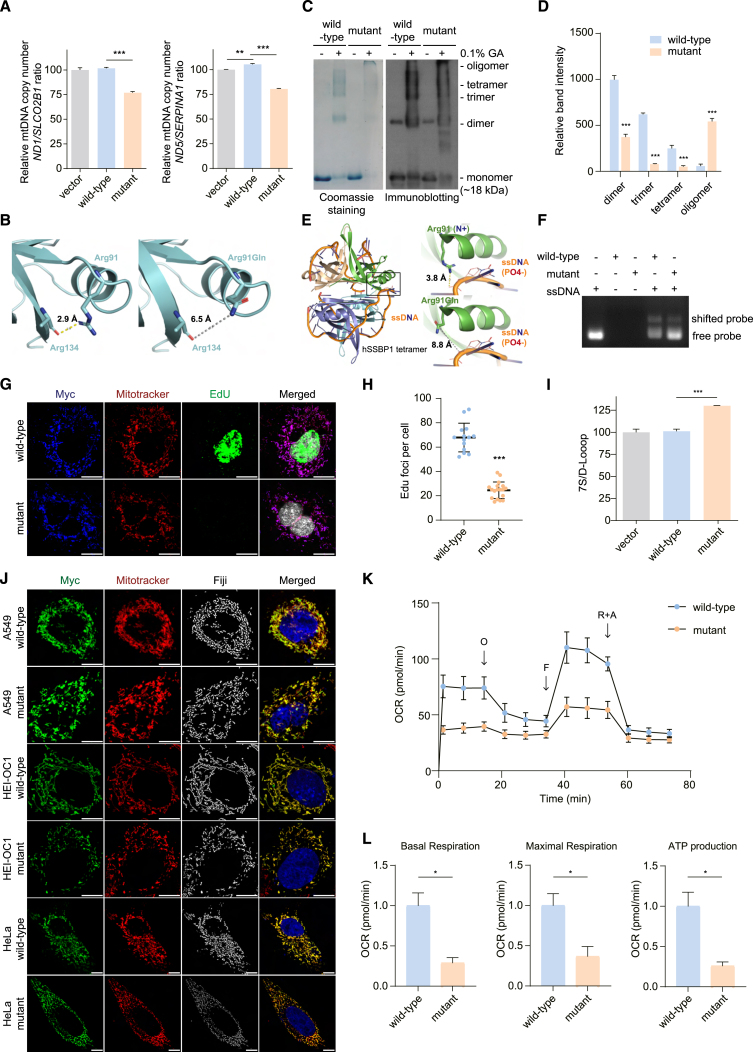
Overexpression of mutant *SSBP1* induces mtDNA depletion and mitochondrial dysfunction in A549 cells through the inhibition of mtDNA replication (A) Quantification of mtDNA was performed using genomic DNA isolated from A549 cells overexpressing *SSBP1*. Relative copy numbers of mtDNA genes *ND1* and *ND5* were measured using quantitative real-time PCR. *SLCO2B1* and *SERPINA* DNA levels were used to normalize the results; ∗∗*p* < 0.01, ∗∗∗*p* < 0.001 (mean ± SEM, *n* = 3 independent experiments). (B) Ribbon diagram showing intrachain interaction of SSBP1 Arg91 with Ala134 (PDB: 3ULL). The Ala134 residue forms a hydrogen bond with the Arg91 residue; however, the interaction with the Ala134 residue was lost with the Arg91Gln mutant. (C and D) Images of Coomassie Brilliant Blue staining (left) and SSBP1 immunoblotting for SSBP1 recombinant proteins after oligomerization. Each immunoblot band intensity was quantified using ImageJ, ∗∗∗*p* < 0.001 (means ± SEM, *n* = 3 independent experiments, unpaired Student’s t test). (E) Ribbon diagram of SSBP1 tetramer structure with aligned ssDNA (PDB: 1EYG). The negatively charged ssDNA interacts with the positively charged Arg91 residue; however, the Arg91Gln residue is predicted not to interact. (F) Electrophoretic mobility shift assay analysis of SSBP1 was conducted using a ssDNA probe at a concentration of 250 nM, which was incubated with 1 μM of both wild-type and mutant SSBP1 tetramers. (G and H) Representative confocal images of A549 cells with *SSBP1* overexpression with EdU incorporation (green). *SSBP1* was stained using Anti-Flag (blue), while mitochondria were stained with MitoTracker (red) (also see Figure S7) and nuclei were stained with DAPI (white). The number of EdU foci per cell was counted manually. ∗∗∗*p* < 0.001 (mean ± SEM, *n* = 14–18, unpaired Student’s t test). Scale bar, 10 μm. (I) 7S DNA was quantified in A549 cells overexpressing *SSBP1* using quantitative real-time PCR. The results were normalized to mtDNA levels; ∗∗∗*p* < 0.001 (means ± SEM, *n* = 3 independent experiments, unpaired Student’s t test). (J) Representative images of dynamics of the mitochondrial network in A549, HEI-OC1, and HeLa cells stained with MitoTracker (red), Flag (green), and DAPI (blue). Images processed with Fiji software, visualized in white. Scale bar, 10 μm. (K) OCR in HeLa cells overexpressing *SSBP1* wild-type and mutant under basal conditions and after injection of oligomycin (O), carbonyl cyanide 4-(trifluoromethoxy) phenylhydrazone (FCCP; F), rotenone (R) and antimycin A (AA). (L) Basal respiration, maximal respiration, and ATP production in HeLa cells were calculated from OCR traces and are reported in the graph; ∗*p* < 0.05 (mean ± SEM, *n* = 5, unpaired Student’s t test).

To explore the molecular consequences of the *SSBP1* c.272G>A mutation, we first assessed the impact of overexpressing Flag-tagged wild-type or p.Arg91Gln mutant protein on mtDNA copy number. By performing a quantitative real-time PCR analysis on the mtDNA-specific genes *ND1* and *ND5* and normalizing it to *SLCO2B1* or *SERPINA1*,^31^ we observed a significant decrease in mtDNA copy number in transiently overexpressed mutants compared with the wild-type protein (Figure 2A). Similarly, both patient-derived fibroblasts and muscle tissue also showed marked reductions in mtDNA copy number (Figure S6). However, in mitochondria isolated from A549 cells, SSBP1 protein was similarly expressed in the p.Arg91Gln mutant and the wild type (Figure S7A). Subsequent quantitative real-time PCR also indicated there was no significant difference in *SSBP1* mRNA between transient overexpression of SSBP1 wild type and the p.Arg91Gln mutant (Figure S7B). We also performed an immunofluorescence assay to visualize the colocalization of SSBP1 with MitoTracker Red-labeled mitochondria. We found a significant overlap between SSBP1-positive Flag signals and mitochondrial networks, confirming that SSBP1 is a mitochondrial protein (Figure S7C). Our findings suggest that the transcription and translation of the SSBP1 p.Arg91Gln mutant is similar to its wild-type counterpart. We, therefore, hypothesized that the subsequent decrease in mtDNA copy number may be due to a functional deficiency.

### *SSBP1* c.272G>A mutation impedes multimer formation and disrupts DNA-binding affinity

In humans, wild-type SSBP1 protein forms a stable tetramer formed from two dimers.^17^ Using the crystal structure of SSBP1 (PDB: 3ULL), we examined the impact of the p.Arg91Gln mutation on protein stability and multimerization. While the Arg91 residue is not involved in interchain interactions, the substitution to Gln91 disrupts the hydrogen bond between the NH^+^ of Arg91 and the C=O of Ala134 carboxylic backbone of the same protomer (Figure 2B). To examine the effect of the mutation in SSBP1 oligomerization, we performed glutaraldehyde crosslinking experiments on wild-type and mutant proteins (Figure 2C), and on whole cell lysates from fibroblast cells (Figure S7D). In contrast with the wild-type protein, which exhibited typical multimer formation including di-, tri-, and tetramers, the mutant showed a reduced capacity to form each of these multimers, with only 37.4%, 12.7%, and 20.5% of dimers, trimers, and tetramers formed, respectively, as compared with wild type. In addition, the mutant protein showed more high oligomeric species, indicating that the mutant protein might form aggregates due to its lower stability.

Next, we tested the binding strength of both wild-type and mutant proteins to ssDNA. During replication, SSBP1 directly binds to single-stranded mtDNA in its tetramer form to prevent the re-annealing of strands and protect against nucleolytic attacks.^15^^,^^32^ Through molecular modeling of the SSBP1-ssDNA complex, we speculated that our mutation would reduce DNA-binding affinity by removing the ionic interaction between the positively charged Arg91 and the negatively charged DNA phosphate backbone (Figure 2E), possibly leading to reduced DNA-binding affinity. Although SSBP1 has been shown to directly bind to DNA,^33^ the molecular interaction between the protein and the DNA is not well understood due to a lack of structural information. We therefore proceeded to examine the DNA-binding affinity of the *SSBP1* mutation. Electrophoretic mobility shift assay (EMSA) demonstrated that our ssDNA probe interacted specifically with wild-type SSBP1 but not the Arg91Gln mutant in a dose-dependent manner, indicating the Arg91Gln mutation significantly decreases DNA binding (Figures 2F and S8). In summary, the *SSBP1* mutation identified in our patient seems to impede multimer formation and disrupt DNA-binding affinity without affecting the abundance of SSBP1 itself. Considering both multimer formation and DNA-binding affinity play pivotal roles in ensuring proper mtDNA maintenance,^34^ we hypothesize that this mutation compromises mtDNA replication efficiency, which has been associated with aberrant mtDNA maintenance.

### *SSBP1* c.272G>A mutation compromises mtDNA replication fidelity and alters mitochondrial network dynamics

To evaluate the effect of the *SSBP1* c.272G>A mutation on the efficiency of mtDNA replication, we performed an EdU incorporation assay to visualize and quantify mtDNA synthesis in our transient overexpression system. We found that, in cells expressing the mutated SSBP1, the co-localization of EdU foci in mitochondria (i.e., foci within the mitochondria) occurred at 36.1% of the amount observed in wild-type cells, indicating that mtDNA replication was reduced (Figures 2G and 2H). Consistent with this result, we also observed a significant rise in 7S DNA levels in cells harboring the mutation (Figure 2I). Considering the role of 7S DNA in the replication machinery,^35^ increased 7S DNA in mutant cells may reflect incomplete mtDNA replication and could be a compensatory response to the decreased replication rate.

The mtDNA replication machinery and mitochondrial maintenance are known to be inter-related.^36^ We subsequently investigated the number of nucleoids co-localizing with mitochondria labeled by MitoTracker Red. Our data revealed a 42% decrease in anti-DNA immunofluorescence in the mutant cells relative to the wild-type cells (Figure S9), which agreed with the observed decrease in mtDNA copy number in mutant SSBP1-transfected cells. Additionally, we visualized the dynamics of mitochondrial network using the MitoTracker Red. We observed increased fragmentation in various cell lines, including A549, HEI-OC1, and HeLa, that overexpress the SSBP1 mutant compared with those expressing the wild-type SSBP1 (Figure 2J). Similarly, A549 cells with the SSBP1 mutant displayed reductions in fragmentation area, perimeter, aspect ratio, form factor, branch length, number of branches, and branch junctions (Figure S10). These observations imply that the *SSBP1* mutation may cause changes in mitochondrial network dynamics due to compromised mtDNA replication and maintenance.

### mtDNA depletion influences bioenergetics in *SSBP1* mutant cells

To explore the ramifications of mtDNA depletion on cellular metabolism, we examined mitochondrial function using Seahorse assays. The oxygen consumption rate (OCR) revealed diminished respiratory function in various cell lines, including HeLa, A549, and HEK293T, that overexpress the SSBP1 mutant compared with those expressing wild-type SSBP1 (Figures 2K and S11). This decrease in the OCR for the mutant cells was consistently noted, with significant decreases in basal respiration, maximal respiration, and ATP production. (Figures 2L and S11). In addition, we confirmed that the expression level of oxidative phosphorylation system (OXPHOS) complex were reduced in cells overexpressing the SSBP1 mutant compared with wild-type SSBP1 (Figure S12). These findings suggest that SSBP1 may contribute to mitochondrial ATP synthesis in addition to its role in mtDNA replication.

### Correction of *SSBP1* mutation using ABE variants to balance editing efficiency and off-target effects

**Figure 3:**
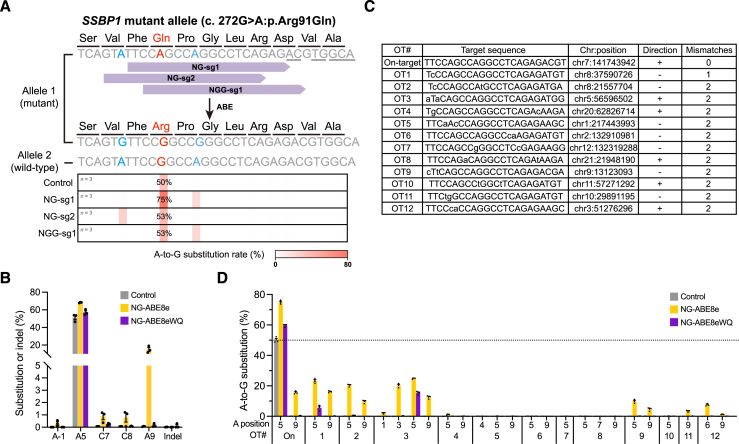
Analysis of sgRNA and ABE variant efficacy for *SSBP1* mutation and their off-target effects (A) Positions and editing efficiencies of sgRNA candidates within the *SSBP1* gene. The red colored A indicates the target nucleotide (c.272G>A) and blue colored A indicates bystander As. A-to-G conversion rate for each nucleotide is shown using heatmaps. Underlined base identifies the PAM of each sgRNA. The editing frequency displayed on heatmap is an average value between three experimental replicates. (B) A-to-G or C-to-other conversion or indel rate after using NG-sg1. The position of base is defined as counting the end distal to the PAM as position 1. (C) Potential sgRNA-dependent off-target sites investigated by Cas-OFFinder software. (D) sgRNA-dependent off-target editing outcomes of ABE-treated patient-derived fibroblast cells. The dashed line indicates 50% frequency because of the heterozygosity of on-target.

To correct the heterozygous *SSBP1* point mutation (c.272G>A:p.Arg91Gln) in patient-derived fibroblasts, we used the Cas9-based editor ABE8e that targeted an NG protospacer adjacent motif (NG-ABE8e) with two different single-guide RNAs (NG-sg1, NG-sg2), or a canonical Cas9-based ABE8e with one sgRNA (NGG-sg1). While the NG-sg2 and NGG-sg1 demonstrated a very poor A-to-G substitution rate (average 53.46% and 53.4%, respectively, including normal allele frequency), NG-sg1 showed the highest A-to-G substitution rate (average 75% including normal allele frequency) (Figure 3A). Therefore, we chose to focus on NG-sg1 to correct the missense mutation. Although NG-ABE8e with NG-sg1 corrected the target A with high frequency, it also showed some bystander editing. These edits included nucleotides near the target A (A5, by counting the end distal to the PAM as position 1), adenines (A-1, A9) and cytosines (C7, C8) at the following frequencies: A-1: 0.29 ± 0.27%; A9: 14.99 ± 3.86%; C7: 0.87 ± 0.44%; and C8: 0.79 ± 0.63% (Figure 3B). Fortunately, the conversion of either bystander A (A-1, A9) to G leads to a silent mutation that does not alter the amino acid sequence of SSBP1. In contrast, bystander C editing (C7, C8) can generate additional missense mutations (Pro92Ser for C7 substitution, Pro92Leu for C8 or both C7, C8 substitution) due to the off-target activities of ABEs.^37^ Although the frequency of bystander C editing was low, we tested a different ABE variant with mutations at V106W and D108Q (NG-ABE8eWQ) that has been shown to minimize bystander C-to-G editing by using a narrower targeting window.^38^ As expected, bystander C editing was reduced; however, this also compromised target A (A5) editing efficiency (average of 57.09% ± 3.17%, including normal allele frequency) (Figure 3B).

We also investigated sgRNA-dependent off-target effects from NG-sg1. We used Cas-OFFinder software to identify 12 potential off-target sites (OT1-OT12) when up to three mismatched bases were allowed^39^^,^^40^ (Figure 3C). Because NG-ABE8e has a relatively wide editing window and high deaminase activity, NG-ABE8e showed substantial off-target editing frequency at OT1-OT3, OT9, and OT11-OT12 (Figure 3D). OT1-OT3 and OT11 are in non-coding regions, whereas OT9 and OT12 are in intron regions within the genes *MPDZ* and *DOCK3*, respectively. Meanwhile, NG-ABE8eWQ showed significantly reduced off-target editing (Figure 3C). These results suggest that NG-ABE8e may present the greatest functional recovery and the risks from bystander editing may be limited due to mostly silent mutations and off-target sites in non-translated regions. In contrast, our use of the safer ABE variant NG-ABE8eWQ also corrected the *SSBP1* mutation, but with reduced efficiency.

### ABEs improve mtDNA copy number and replication efficiency in patient-derived fibroblasts

We next evaluated the efficacy of these ABE variants (NG-ABE8e and NG-ABE8eWQ) to restore mtDNA copy number and mitochondrial replication. We performed genome editing in patient-derived fibroblasts and compared them with two control cell lines from healthy individuals without *SSBP1* mutations (control 1), and parental control cells (control 2). One concern with assessing mitochondrial function in cell culture can be the potential confounding effects from cellular aging and senescence.^41^^,^^42^ Our observations showed that patient fibroblast cells displayed substantial cellular senescence (β-galactosidase staining and swelling) after passage 7, whereas control and genome-edited cells had a limited number of senescent cells (Figure S13). We, therefore, used fibroblast cells between passages 4 and 7 in our genome-editing experiments to limit these age-dependent effects.

**Figure 4:**
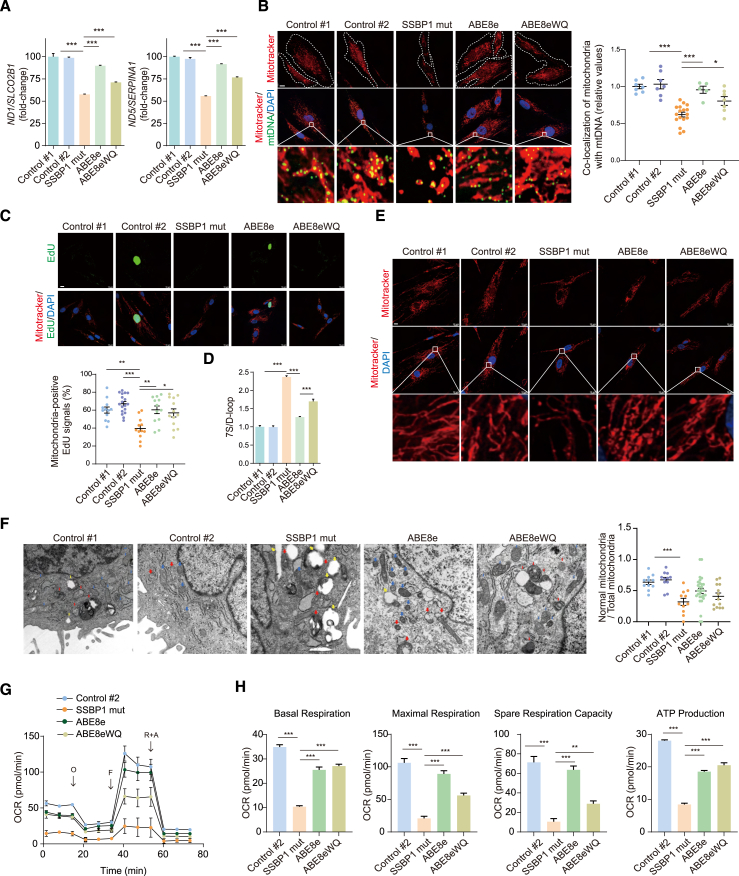
Functional recovery of mitochondria in patient-derived fibroblasts using ABEs (A) Genomic DNA was isolated from the fibroblast cell lines and subjected to quantitative real-time PCR for *ND1* and *ND5*. *SLCO2B1* or *SERPINA* was used to normalize the results; ∗∗∗*p* < 0.001 (mean ± SEM, *n* = 3 independent experiments). (B) Representative confocal images showed control, patient-derived, and genome-edited fibroblasts stained with MitoTracker (red) and anti-DNA (green). The boxes in the merged images point to the enlarged sections displayed at the bottom of each panel. The relative amounts of DNA that co-localized with MitoTracker were quantified; ∗*p* < 0.05, ∗∗∗*p* < 0.001 (means ± SEM, *n* = 5–17, one-way ANOVA followed by the Bonferroni *post hoc* test). Scale bar, 10 μm. (C) Same as (B), except that immunofluorescence with EdU incorporation. The co-localization of MitoTracker (red) and EdU (green) foci was employed to confirm the replication of mtDNA. The EdU incorporation into the mitochondria of different fibroblast cell lines was calculated and statistically analyzed; ∗*p* < 0.05, ∗∗*p* < 0.01, ∗∗∗*p* < 0.001 (mean ± SEM, *n* = 12–18, one-way ANOVA followed by the Bonferroni *post hoc* test). Scale bar, 10 μm. (D) Quantification of 7S DNA within the genomic DNA from fibroblast cell lines by quantitative real-time PCR. The mtDNA level was used to normalize the result (mean ± SEM, *n* = 3 independent experiments, one-way ANOVA followed by the Bonferroni *post hoc* test). (E) Representative confocal images depict mitochondrial fission in fibroblast cell lines. Mitochondrial network formation was assessed through MitoTracker staining (red). The boxes in the merged images indicate the enlarged sections shown at the bottom of each panel. Scale bar, 10 μm. (F) Transmission electron microscopy images of mitochondria with normal (blue arrow), large vacuole (yellow arrow), and abnormal (red) morphology in fibroblast cell lines. The relative proportion of normal mitochondria within the total mitochondria population was calculated; ∗∗∗*p* < 0.001 (mean ± SEM, *n* = 7–29, one-way ANOVA followed by the Bonferroni *post hoc* test). (G) OCR in fibroblasts under basal conditions and after injection of oligomycin (O), carbonyl cyanide 4-(trifluoromethoxy) phenylhydrazone (FCCP; F), rotenone (R), and antimycin A (AA). (H) Alterations in basal respiration, maximal respiration, and ATP production in fibroblasts were derived from OCR traces; ∗∗*p* < 0.01, ∗∗∗*p* < 0.001 (mean ± SEM, *n* = 3–6 one-way ANOVA followed by the Bonferroni *post hoc* test).

First, we examined SSBP1 protein expression in our fibroblast cell lines. Consistent with the data from the overexpression system, the levels of SSBP1 protein seemed to be comparable between the groups (Figure S14). We then quantified the mtDNA copy number using quantitative real-time PCR for mtDNA-encoded *ND1* and *ND5* and normalized it to *SLCO2B1* or *SERPINA1*. The mtDNA copy number in patient fibroblast cells was significantly decreased compared with control fibroblasts, whereas the genome-edited cells consistently demonstrated a substantial recovery in mtDNA copy number (Figure 4A). On average, the mtDNA content in genome-edited cells increased by 59.9% with NG-ABE8e and 30.2% with NG-ABE8eWQ. In line with this, the relative number of nucleoids co-localizing with mitochondria was markedly decreased in patient fibroblasts but was significantly ameliorated in the genome-edited cells (Figure 4B). In the edited fibroblasts, the nucleoid ratio increased by 1.5-fold with NG-ABE8e and 1.3-fold with NG-ABE8eWQ compared with patient cells. These levels correspond with 94.0% and 79.1%, respectively, of those observed in control cells (Figure 4B).

We subsequently evaluated whether ABEs enhance mtDNA replication efficiency in patient fibroblasts by visualizing the EdU signal colocalized with mitochondria, which would suggest restoration of mtDNA copy number. Patient fibroblasts exhibited a significant decrease in the number of EdU foci that colocalized with mitochondria (Figure 4C). In contrast, genome-edited cells with NG-ABE8e and NG-ABE8eWQ demonstrated an increase in these EdU foci by 94.8% and 89.4%, respectively, compared with control fibroblasts (Figure 4C). Consistent with this observation, the elevated 7S DNA in edited patient fibroblasts was decreased by 47.0% or 28.0% when using NG-ABE8e or NG-ABE8eWQ, respectively, relative to patient fibroblasts (Figure 4D). The improvement in mtDNA replication fidelity through base editing, coupled with compensatory adjustments in 7S DNA levels, suggests that mutant SSBP1 may have a causal role in mtDNA depletion disease. These results imply that base editors-based gene therapies may be able to restore replication and improve mtDNA copy numbers, which in turn may contribute to the recovery of compromised mitochondrial function and abnormal dynamics observed in patient cells.

### ABE variants improve bioenergetics and mitochondrial dynamics

Next, we compared the mtDNA integrity among control groups, our patient-derived fibroblast, and ABE8e-modified patient-derived fibroblasts. Similar to our overexpression system, mtDNA depletion also compromised the mitochondrial network in our patient-derived fibroblasts, as characterized by pronounced fragmentation and mitochondrial shortening (Figure 4E). Interestingly, NG-ABE8e- or NG-ABE8eWQ-treated patient-derived fibroblasts exhibited significant improvements in these dynamics (Figure 4E). To further elucidate the impact of ABEs on mitochondrial morphology and abundance, we examined the ultrastructural features of mitochondria by transmission electron microscopy (Figure 4F). Alterations in mitochondrial fusion and fission have been associated with changes in mitochondrial shape and number, particularly inner membrane (i.e., cristae) remodeling during apoptosis.^43^ In this study, abnormal mitochondrial morphology was defined as either vesicular or swollen forms. Normal mitochondria are characterized by dense staining of the cristae alongside an intact outer membrane. In contrast, vesicular mitochondria display morphological features where the cristae are separated from the inner membrane, with circular or rounded cristae distributed throughout the mitochondrial body. The swollen mitochondria exhibit fragmented or disorganized cristae, with large vacuole features.^43^ In patient fibroblasts, the overall mitochondrial ultrastructure was abnormal, showing vesicular, swollen, or vesicular-swollen forms (Figure 4F). Furthermore, the patient-derived cells exhibited a substantial decrease in the proportion of normal mitochondria, corresponding with 48.9% of the control fibroblast levels. In contrast, the edited cells displayed a notable increase in the proportion of normal mitochondria, which corresponded to 75.5% and 62.6% of control fibroblast levels for NG-ABE8e and ABE8eWQ, respectively, with no statistically significant difference between the two base editors (Figure 4F).

We next investigated the impact of base editors on SSBP1-dependent bioenergetics. The OCR assay showed a markedly reduced respiratory capacity in patient fibroblasts compared with controls (Figure 4G). In contrast, treatment with either of the two ABEs significantly enhanced basal respiration (*p* < 0.001 for NG-ABE8e; *p* < 0.001 for NG-ABE8eWQ), maximal respiration (*p* < 0.001 for NG-ABE8e; *p* < 0.001 for NG-ABE8eWQ), spare respiration capacity (*p* < 0.001 for NG-ABE8e; *p* = 0.029 for NG-ABE8eWQ), and mitochondrial ATP production (*p* < 0.001 for ABE8e; *p* < 0.001 for ABE8eWQ), compared with patient cells (Figure 4H). In the edited cells, both ABEs exhibited enhanced basal respiration, with values of 2.46-fold for NG-ABE8e and 2.61-fold for NG-ABE8eWQ when compared with patient cells. This corresponds to 73.0% and 77.6% of the control cell levels, respectively. These changes in respiratory capacity led to increased ATP synthesis by OXPHOS, with increases of 2.01-fold for NG-ABE8e and 2.11-fold for NG-ABE8eWQ when compared with patient cells (Figure 4H). These values represent 66.1% and 73.1% of the control cell levels, respectively. Additionally, the spare respiratory capacity in the edited cells showed a marked increase (3.12-fold for NG-ABE8e and 2.08-fold for NG-ABE8eWQ) in comparison with patient cells (Figure 4H). These increases correspond with 89.2% and 40.5% of control cell levels, respectively. Such an enhanced spare respiratory capacity in the edited cells suggests that their mitochondria may have the ability to respond to increased energy demands or metabolic stress. In summary, these findings suggest that, even when a base editor was less efficient at correcting a mutation (here *SSBP1*), it could still substantially improve mitochondrial function, including bioenergetics and mitochondrial dynamics.

### *SSBP1* disease-causing mutations are suitable for base editing

**Figure 5:**
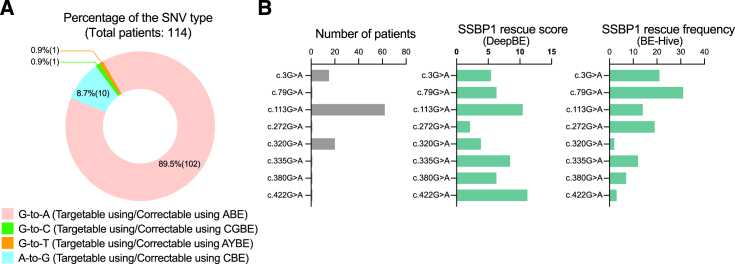
Characterizations of *SSBP1* mutations in patients and predictions of base editing efficacy (A) Type and distribution of the 114 single nucleotide variations (SNVs) reported in the literature for *SSBP1*. Among them, 89.5% display G-to-A single base transitions, whereas 8.7% manifest A-to-G transitions, indicating the dominance of single base transition mutations. (B) Assessment the number of patients and the corresponding *SSBP1* rescue score. A total of 8 G-to-A mutations associated with mtDNA depletion syndrome for potential base editing applications. Predictions were carried out using the DeepBE and BE-Hive software. The *SSBP1* rescue score (or frequency) indicates that the mutation is corrected without any other amino acid mutations.

In our literature review (Table S2), we noted that all of the documented *SSBP1* mutations with mitochondrial phenotypes were single nucleotide variations. Therefore, base editors could potentially target all of these mutations. Of all patients with *SSBP1* mutations, 89.5% and 8.7% have either G-to-A or A-to-G single base transition mutations, which can be corrected by ABE and CBE, respectively. The remaining 1.8% of patients have either G-to-T or G-to-C single base transversion mutations (Figure 5A), which could potentially be corrected by more recently developed base editors. The A-to-Y base editor, described by the Yang group, can induce A-to-C or A-to-T substitution to edit G-to-T mutations.^44^ For G-to-C mutations, the C-to-G base editor, characterized by the Joung group, can be used.^45^ Although all *SSBP1* disease-causing mutations can theoretically be corrected using a variety of base editors, the majority of pathogenic *SSBP1* mutations are G-to-A transitions that can be corrected by ABE. We, therefore, used the DeepBE and BE-Hive machine learning models, which can predict base editing efficacy in silico,^46^ to simulate the potentially efficacy of ABEs to correct this type of *SSBP1* mutation. The prediction was performed based on the ABE8eW with diverse Cas9 variants for DeepBE and ABEmax for BE-Hive. The results from DeepBE showed all other mutations examined a more favorable base editing rescue score in comparison to c.272G>A, which was the original mutation we identified in this study (Figure 5B). In case of the BE-Hive, the results are slightly different from the DeepBE results (Figure 5B). These differences can arise from variations in both TadA version and nickase Cas version while performing the predictions using each model. According to these two prediction models, most of the mutations can be covered by ABE with high frequency. However, targets with low predicted efficacy, such as c.320G>A, c.380G>A, or c.422G>A, require a deliberate decision of sgRNA and ABE variant. Given the substantial improvement in mitochondrial function we observed after correcting the *SSBP1* c.272G>A mutation, these predictions indicate that all known *SSBP1* G-to-A mutations may be suitable candidates for base editing-based gene therapy in clinical applications. Notably, the most prevalent mutation reported to date (c.113G>A) is predicted to have enough editing efficiency using an NG-Cas9 based ABE, which suggests that a large percentage of patients could benefit from this strategy.

## Discussion

Mitochondrial replication plays an essential role in safeguarding the integrity and abundance of mtDNA to regulate cellular energy synthesis. Disruptions or anomalies in this complex process can lead to mitochondrial disorders that impair cellular vitality and function. Here, we report a novel mutation in *SSBP1* (c.272G>A:p.Arg91Gln) that impacted its multimer formation and DNA-binding affinity in purified proteins. This reduced multimer formation was similarly observed in lysates from patient-derived cells. Subsequently, these changes significantly reduced mtDNA replication and altered mitochondria dynamics, resulting in mtDNA depletion and mitochondrial dysfunction. These findings were consistent across both *SSBP1* mutant overexpression system and patient-derived fibroblasts. Collectively, our findings replicate previous reports on the molecular consequences of *SSBP1* mutations and their association with mtDNA depletion-related mitochondrial disorders.

Mutations in *SSBP1* are associated with a variety of tissue-specific phenotypes, but primarily present as optic atrophy.^15^^,^^16^ *In vivo* studies in *ssbp1*-null zebrafish mirrored the optic atrophy observed in patients with dominant *SSBP1* mutations.^16^ Patients can also exhibit a spectrum of additional neurological symptoms. Despite genotype-phenotype variabilities among different *SSBP1* mutations, reports of sensorineural deafness are particularly prevalent in the literature (Figure S1). It is well known that clinical manifestations can overlap among various replisome components, including SSBP1, due to their interaction operative in the replisome and shared mechanisms in mtDNA replication and maintenance.^11^^,^^12^ Sensorineural deafness is one of the overlapping phenotypes in mutations in mtDNA replisome machinery. However, the underlying mechanism of how hearing loss is linked to *SSBP1* mutations remains poorly understood. We observed that SSBP1 is expressed in the mouse cochlea, particularly its sensory hair cells, which requires substantial energy for mechanoelectrical sound transduction, ion recycling, and homeostasis. Mitochondria regulate the function of hair cells and participate in apoptosis pathways that respond to intrinsic and extrinsic factors.^47^ Thus, mtDNA depletion and subsequent mitochondrial dysfunction in cochlear hair cells may contribute to the development of sensorineural deafness in patients with *SSBP1* mutations.

To correct patient-derived cells harboring a heterozygous missense mutation (c.272G>A:p.Arg91Gln), we tested two ABE variants. The NG-ABE8e variant exhibited the greatest editing efficacy and demonstrated optimal recovery of mitochondrial function. This can be attributed to the fact that most bystander editing results in silent mutations and that the off-target sites are not located within the open reading frame. The NG-ABE8eWQ had a more specific editing window and, therefore, displayed a diminished editing efficacy, but still showed substantial functional recovery. Importantly, although the mutations introduced into the NG-ABE8eWQ reduced overall editing capacity,^48^ it also led to notably fewer off-target effects. The reason for diminished both on-target editing efficacy and undesired editing efficacy can be interpreted as the same tendency with ABE8eW invented by Liu’s group.^48^ The ABE8e shows significantly high editing efficacy and a wide editing window, which also induces a high frequency of undesired editing such as sgRNA-dependent off-target, RNA off-target, or bystander C editing. Therefore, Liu’s group applied TadA-V106W mutation to the ABE8e (ABE8eW) for decreasing RNA off-target.^48^ Overall, the ABE8eW shows reduced RNA off-target editing efficacy but also relatively decreased on-target editing efficacy. In case of the ABE8eWQ, it shows further minimized RNA off-target editing and bystander C editing without any compromised on-target editing efficacy compared with the ABE8eW. From a perspective of functional restoration, the NG-ABE8e shows the best performance, but, considering the safety issue, the NG-ABE8eWQ variant may be a better therapeutic candidate because it also shows meaningful functional restoration. As base editing makes its way into the clinic, our research offers evidence that base editing-based gene therapies are a promising treatment option for *SSBP1* mutations and other nuclear genomic mutations associated with mtDNA maintenance diseases.

Our findings indicate that correction of disease-causing pathogenic alleles via gene editing could facilitate the recovery of mitochondrial function. To our knowledge, this report is the first of the functional recovery of mitochondria diseases through base editing-based gene therapies. Base editor-treated patient-derived fibroblast displayed the improved mtDNA replication fidelity and the normalized mitochondrial morphology including the mitochondrial dynamics and ultrastructures. The morphological restoration of the mitochondria might be attributed to the increase of mtDNA abundance, modifications in mitochondrial dynamics related to fusion and fission, or a combination of both. Alternatively, base editing might moderate cellular senescence (Figure S9), which possibly avert the mitochondrial dysfunction caused by *SSBP1* mutations.

In summary, we identified a novel *SSBP1* mutation and its causal relationship to mitochondrial disease. We also demonstrated gene editing could significantly enhance mtDNA replication fidelity and mtDNA copy number by correcting the mutation using two different base editors. Although base editing-based therapeutics have a strong potential for mitochondrial diseases, substantial challenges persist before they can be clinically applied. Efficacy and specificity can be of particular concern in mitochondria diseases with multisystemic involvement. Nevertheless, several of the phenotypes-associated *SSBP1* mutations seem to be tissue specific, primarily including optic atrophy and sensorineural deafness. The eye and ear sensory organs are strong candidates for gene therapies and genome editing due to their unique properties, such as a small, enclosed compartment, immune privilege, and accessibility via established injection approach.^49^^,^^50^ Therefore, we believe that base editing-based gene therapies may be a feasible strategy for correcting *SSBP1* mutations. In a clinical landscape where most patients with mitochondrial diseases lack a definitive treatment, the ability to restore mitochondrial function through base editing may be an attractive option for physicians and patients.

## Materials and methods

### Study subjects

We included the participants within the Hereditary Hearing Loss Clinic of the Center for Rare Diseases, Seoul National University Hospital, South Korea. All procedures were approved by the Institutional Review Board of Seoul National University Hospital (no. IRB-H-0905-041-281 and IRB-H-2202-045-1298). Demographic data and clinical phenotypes were retrieved from electronic medical records.

### Molecular genetic testing

Genomic DNA was isolated from peripheral blood samples using the Chemagic 360 instrument (PerkinElmer, Baesweiler, Germany). In the initial phase (step 1), we examined 22 mutations across 10 established deafness genes (*GJB2*, *SLC26A4*, *TMPRSS3*, *CDH23*, *OTOF*, *TMC1*, *ATP1A3*, *MPZL2*, *COCH*, and *12S rRNA*) through genotyping techniques such as custom capillary sequencing and U-TOP HL Genotyping Kits.^51^^,^^52^ During the subsequent phase (step 2), targeted sequencing was achieved using whole-exome sequencing (WES). For this, target regions were captured employing the SureSelectXT Human All Exon V5 kit for WES (Agilent Technologies, Santa Clara, CA, USA). Libraries were constructed in line with the manufacturer’s protocol and subjected to paired-end sequencing on the NovaSeq 6000 system (Illumina, San Diego, CA, USA). In the concluding phase (step 3), patients not yet diagnosed underwent WGS followed by comprehensive bioinformatic analysis and curation. DNA libraries were assembled using the TruSeq DNA PCR-Free Library Prep Kits (Illumina) and sequenced on the Illumina NovaSeq6000 platform with an average coverage depth of 30×. Resultant genome sequences were mapped to the human reference genome (GRCh38) utilizing the BWA-MEM algorithm. PCR duplicates were eliminated with SAMBLASTER. Primary mutation detection for base substitutions and short indels was conducted using HaplotypeCaller2 and Strelka2, correspondingly. Subsequently, mutations underwent sieving, Mendelian inheritance patterns were assessed, and potential *de novo* mutations, along with their predicted impacts, were identified. The parental samples were used for Sanger sequencing to determine the phasing. Medical geneticists made the final assessment of mutation pathogenicity, according to ACMG/AMP guidelines.^28^

### Purification of recombinant SSBP1 proteins

To purify the wild-type and its mutants, *E. coli* strain BL21 (DE3) cells (Agilent) were cultured at 37°C until the OD at 600 nm reached 0.6. Subsequently, 0.5 mM isopropyl-β-d-thiogalactopyranoside was introduced to induce SSBP1 expression, and cells were allowed to grow 16 h at 16°C. Upon completion of expression, the cells were collected by centrifugation and lysed using sonication in a lysis buffer (50 mM Tris buffer pH 7.0 containing 250 mM NaCl, 4 mM MgCl_2_, 25 μg/mL DNase I, 25 μg/mL RNase A, and a protease inhibitor cocktail). The resulting supernatant was applied to a 5 mL HiTrap IMAC FastFlow column (GE Healthcare Life Sciences, Chicago, IL, USA) utilizing the ÄKTA FPLC system (GE Healthcare Life Sciences). The column was washed with five column volumes of washing buffer (50 mM Tris pH 7.0 with 250 mM NaCl), and elution was carried out using an imidazole step gradient. After elution, the proteins were subjected to dialysis, followed by size exclusion chromatography. These dialyzed proteins were loaded onto a HiLoad 16/600 Superdex 200 pg by fast protein liquid chromatography. To each fraction, 10% glycerol was added, and the proteins were stored at −80°C.

### Molecular modeling and structure analysis

The crystal structure of the wild-type SSBP1 protein was obtained from the Protein DataBank (PDB: 3ULL). The mutagenesis was generated using PyMOL software (ver. 2.5.2). Additionally, the model structure of the SSBP1-ssDNA complex was generated and visualized by superimposing the SSBP1 crystal structure (3ULL) to the *E*. *coli* ssb-ssDNA complex structure (1EYG). Inter- and intrachain changes, including hydrophobic, hydrogen bonds, and salt bridge interactions, were compared with predict the structural effects of *SSBP1* mutation. All graphical illustrations were produced using PyMOL software (ver. 2.5.2) (PyMOL Molecular Graphics System ver. 2.0, Schrödinger Inc., New York, NY, USA).

### SDS-PAGE and immunoblotting

Cultured cells were washed twice with PBS, and whole-cell lysates were prepared in RIPA buffer supplemented with a protease inhibitor cocktail. For SDS-PAGE, lysates mixed with NuPAGE 4× LDS sample buffer (NP0007, Invitrogen, Carlsbad, CA, USA) and denatured at 85°C for 10 min separated proteins by SDS-PAGE were transferred to a polyvinylidene difluoride membrane. The membranes were blocked with 5% nonfat milk in TBS-T solution and incubated with the primary antibodies. The membranes were then washed with TBS-T solution several times and followed by a horseradish peroxidase-conjugated anti-rabbit IgG or anti-mouse IgG antibody. After antibody incubation, protein band were detected by chemiluminescence reagent (RPN2106, Cytiva, Marlborough, MA, USA). Antibodies used in this study are summarized in Table S4.

### Electrophoretic mobility shift assay

The electrophoretic mobility shift assay (EMSA) for SSBP1 was conducted following previously established methods.^53^^,^^54^ Briefly, we utilized custom-synthesized ssDNA oligonucleotides (Table S3), provided by Cosmo Genetech (Seoul, Korea). In the experimental procedure, varying concentrations of both wild-type and mutant SSBP1 recombinant proteins were incubated with a known quantity of single-stranded oligonucleotide probes in a binding buffer (composed of 50 mM Tris pH 7.5, 10% glycerol, and 150 mM NaCl) for a duration of 30 min at room temperature. Subsequently, a loading buffer (Dyne LoadingSTAR, DYNE BIO, Gyeonggi-do, Korea) was added, and the prepared samples were loaded onto 2% agarose gels. Electrophoresis was carried out in 1× Tris-borate-EDTA buffer for 45 min at 100 V. Following electrophoresis, the gels were visualized using UV light.

### Mammalian cell cultures and transient expression

A549 cells were grown in RPMI 1640 supplemented with 10% of FBS, 100 U/mL penicillin, 100 μg/mL streptomycin, and 2 mM L-glutamine. A human *SSBP1* cDNA clone (RC215106) was purchased from Origene (Rockville, MD, USA). The SSBP1 p.Arg91Gln mutation plasmid was generated utilizing the QuickChange mutagenesis method.^55^ For transient overexpression, cells were transfected with 2 μg total plasmid DNA in a six-well culture plate (>70%–80% confluence of cells) for 24 h using Lipofectamine 3000 (L3000001, Invitrogen), according to the manufacturer’s guidelines.

### Genomic DNA and RNA isolation followed by quantitative real-time PCR

Genomic DNA was purified from either A549, fibroblasts, and muscle tissue using Puregene DNA purification kit (158026, Qiagen, Hilden, Germany). Concurrently, total RNA was isolated cells using TRIzol Reagent (15596018, Thermo Fisher Scientific, Waltham, MA, USA). cDNA samples were prepared using one microgram of total RNA by reverse-transcription PCR (Accupower RT-pre-mix, Bioneer, Daejeon, Korea). Quantitative real-time PCR reactions were carried out using either the genomic DNA or cDNA, SYBR qPCR master mixture (RT501M, Enzynomics, Daejeon, Korea) as the reporter dye, and 10 pM primers to detect mRNA expression of specific genes. Primer sequences used in this study are summarized in Table S3.

### Immunocytochemistry and EdU labeling assay

For immunofluorescence microscopy, cultured fibroblast or A549 cells on a cover glass were transfected with 1–2 μg of total plasmid DNA for 24 h. The cells were fixed with 4% paraformaldehyde, then washed with 0.1% Tween 20 in PBS. Next permeabilized with 0.5% Triton X-100, 1% BSA in PBS. And then the cells were incubated with the primary antibody and Alexa Fluor-conjugated secondary antibody. The cells were mounted with 4′,6′-diamidino-2-phenylindole (DAPI)-containing mounting medium (ab104139, Abcam, Cambridge, UK). Confocal images were captured by a laser scanning confocal microscope (Leica STELLARIS 8, Upright). For EdU labeling (C10337, Invitrogen), EdU was added to the culture media at a concentration of 100 μM and incubated for 90–120 min. Subsequently, Alexa Fluor 488 azide was used for detection. After EdU labeling, cells were treated with MitoTracker Red (Invitrogen, M7512) to stain the mitochondria. For quantification, EdU foci that were positive for MitoTracker Red were counted in over 10 individual images for each cell line.

### Mitochondrial networks analysis

Mitochondrial networks were analyzed utilizing the Mitochondrial Network Analysis toolset integrated within the Fiji distribution of ImageJ.^56^ Initially, images were cropped to focus exclusively on individual cells. Subsequent processing involved a series of steps aimed at enhancing image quality and reducing noise. Background subtraction was performed using a rolling radius of 1.25 to eliminate ambient noise. This was followed by the application of a Sigma filter with a radius of 3.35 μm, specifically designed to minimize residual noise. To improve visibility in underexposed regions without increasing noise, the enhance local contrast feature was adjusted with a maximum slope of 1.8. Additionally, gamma correction was applied at a value of 0.80 to fine-tune the brightness and contrast in these regions. For image thresholding, the auto threshold function was employed, setting parameters with a block size of 1.45 μm and a C-value of 5, to ensure optimal differentiation of mitochondrial structures. The processed images were then binarized and skeletonized, preparing them for quantitative analysis. The "Analyze Skeleton" plugin in ImageJ was used to evaluate the structural integrity and connectivity of the mitochondrial networks. A minimum of six images were analyzed to ensure the statistical robustness of the findings. This methodical approach allowed for precise and detailed assessments of mitochondrial morphology and network dynamics within cells,^57^ contributing valuable insights into mitochondrial function and health.

### OCR

The intact cellular OCR and extracellular acidification rate (ECAR) were measured in real time using the Seahorse XF96 Extracellular Flux Analyzer (Seahorse Bioscience, North Billerica, MA, USA), according to manufacturer’s protocol. Briefly, 8.0 × 10^3^ of fibroblast, A549, HEK293T, and HeLa cells were seeded into 96-well Seahorse microplates in 100 μL growth medium and incubated at 37°C in 5% CO_2_ for 24 h and the calibrator plate was equilibrated in a non-CO_2_ incubator overnight. Before starting the test, cells were washed twice with assay running media (unbuffered DMEM, 25 mM glucose, 1 mM glutamine, 1 mM sodium pyruvate) and equilibrated in a non-CO_2_ incubator. Once the probe calibration was completed, the probe plate was replaced by the cell plate. The protocol was optimized and gave the measurement of OCR and ECAR simultaneously. Totally, the assay protocol incorporated four compounds injection, which could be applied to modulate mitochondrial function to determine the mitochondrial parameters, including basal respiration, maximal respiration, and ATP production. The analyzer plotted the value of OCR and the corresponding ECAR followed by injection of the compounds sequentially as follows: oligomycin (1 μM), an inhibitor of ATP synthase which leads to exhibit maximal glycolysis metabolism; followed by exposure of carbonyl cyanide p-(trifluoromethoxy) phenylhydrazone (1 μM), the uncoupler of ETC and OXPHOS which induces the peak oxygen consumption to evaluate the oxidative metabolism indirectly; by addition of ETC inhibitor rotenone and the complex III inhibitor antimycin A to uncover the part of non-mitochondrial respiration at a final concentration of 1 μM.

### Fibroblast cell culture

The fibroblast culture was conducted following the methods described by Vangipuram et al.^58^ Under local anesthesia, a 4-mm round skin biopsy was collected from the donor’s designated area and immediately placed in a 1-mL tube with 1 mL PBS. The biopsy was then transferred to a sterile 6-cm culture dish using sterile forceps and dissected into 9–12 uniformly sized pieces with sterile scalpel blades or scissors. Using pointed sterile forceps, each biopsy piece was placed into individual wells of a 12-well plate containing 200 μL complete DMEM supplemented with 20% fetal bovine serum, ensuring proper attachment to the bottom of the well. The plate was incubated at 37°C and monitored daily for the first week, with up to 50 μL of media added every 2 days to compensate for evaporation. When the fibroblasts formed a confluent monolayer, they were harvested for further expansion.

### Electroporation and cell lysis

Patient-derived fibroblasts (100,000 cells/transfection) were transfected using a Neon transfection system 10 μL kit (Thermo Fisher Scientific, MPK1025) with following parameters: Voltage, 1,600 V; width, 20 ms; number, 1. The fibroblasts were transfected with 900 ng of ABE-encoding plasmid and 300 ng sgRNA-encoding plasmid. After 72 h from electroporation, one-half of the cells were harvested for next-generation sequencing and the other one-half of the cells were maintained for verifying functional recovery. Cell pellets were resuspended in proteinase K extraction buffer containing 40mM Tris-HCl [pH8.0], 1% Tween 20, 0.2 mM EDTA, 10 mg proteinase K, 0.2% Nonidet P-40 (VWR Life Science, Radnor, PA, USA, 97064-730). After resuspension, incubation was performed at 60°C for 15 min and 98°C for 5 min

### High-throughput DNA sequencing

PCR amplification was carried out with primers containing sequencing adaptors using KOD-Multi & Epi (TOYOBO, KME-101) according to the manufacturer’s instructions. After the first PCR amplification, 1 μL of the first PCR products were amplified again with primers containing sequencing barcodes of TruSeq HT Dual index system (Illumina). Then, the second PCR products were purified using Expin PCR SV mini (GeneAll, Seoul, Korea) and sequenced using a Miniseq sequencing system (Illumina). The sequencing results were analyzed using BE-Analyzer.^59^ Analysis of sequencing data was mostly carried out using the computing server at the Genomic Medicine Institute Research Service Center.

### Transmission electron microscopy

Cells were fixed in a 2.5% glutaraldehyde in PBS buffer 48 h at 4°C and washed in PBS buffer 10 min. After that post-fixed in a 1% osmium tetroxide for 2 h at room temperature. After incubation, washed two times in PBS buffer. After that, treat ethanol solutions (30%–100%) for dehydration and embedded in EmBed 812. Ultrathin sections were used for making sample blocks. Staining the sections with uranyl acetate and lead citrate and examined in a JEOL, JEM1400 Flash transmission electron microscope at 80 kV.

### Statistical analysis

We conducted statistical analysis and generated graphs using GraphPad Prism 8.0.1 (GraphPad Software, La Jolla, CA, USA). Immunoblots and gel images were quantified using ImageJ software. Specific experiment details can be found in the figure legends. All experimental data is presented as mean ± SEM. Statistical significance was determined for comparisons with a *p* value <0.05, denoted as ∗*p* < 0.05; ∗∗*p* < 0.01; and ∗∗∗*p* < 0.005.

## Data and code availability

The authors declare that the data supporting the findings of this study are available within the paper and in the Supplemental Materials. Should any raw data files be needed in another format, they are available from the corresponding author upon request.

## Supplemental information

Document S1. Figures S1–S14 and Tables S2–S4

Table S1. Summary of reported SSBP1 mutations, including this study, and its pathogenicity prediction analysis

Document S2. Article plus supplemental information
