# Supplementary material for: Discovery of novel disease-causing mutation in SSBP1 and its correction using adenine base editor to improve mitochondrial function
Source: Mol Ther Nucleic Acids. 2024 Jun 17;35(3):102257. doi: 10.1016/j.omtn.2024.102257 (PMC11299580; doi:10.1016/j.omtn.2024.102257)
Supplement: Document S1. Figures S1–S14 and Tables S2–S4 [file mmc1.pdf]

OMTN, Volume *35*

Supplemental information

Discovery of novel disease-causing mutation in *SSBP1* and its correction using adenine base editor to improve mitochondrial function

Ju Hyuen Cha, Seok-Hoon Lee, Yejin Yun, Won Hoon Choi, Hansol Koo, Sung Ho Jung, Ho Byung Chae, Dae Hee Lee, Seok Jae Lee, Dong Hyun Jo, Jeong Hun Kim, Jae-Jin Song, Jong-Hee Chae, Jun Ho Lee, Jiho Park, Jin Young Kang, Sangsu Bae, and Sang-Yeon Lee

Supplemental Methods

Molecular genetic testing

The genetic testing approach was organized into a sequential stepwise approach[1-5].

Immunohistochemistry

To confirm SSBP1 protein expression in the inner ear, immunocytochemistry was performed in the cryostat section of a 4-week-old C57BL/6J mouse. The sectioned samples were washed three times with PBS and blocked for 30 minutes with 4% BSA and 0.01% Triton-X dissolved in PBS. Blocked samples were stained with anti-SSBP1 (1:400; Proteintech, Cat#12212-1-AP) and labeled with anti-rabbit secondary antibody (1:500; Invitrogen, Cat#A32732). Stained samples were mounted with mounting media with DAPI (Abcam, Cat#ab104139). Stained images and DIC images were taken by Leica STELLARIS 8 confocal microscope.

mtDNA panel sequencing and MLPA

For mitochondria panel sequencing, DNA was extracted from peripheral blood samples using the Chemagic 360 instrument (Perkin Elmer, Baesweiler, Germany). The complete human mitochondrial genome was amplified in two overlapping fragments: fragment I (spanning 9,289 bp), and fragment II (spanning 7,626 bp). Fragment 1 was amplified using the primer pair hmtF1 569 (5′-AACCAAACCCCAAAGACACC-3′) and hmtR1 9819 (5′-GCCAATAATGACGTGAAGTCC-3′), and fragment II was amplified using the primer pair htmF2 9611 (5′-TCCCACTCCTAAACACATCC-3′) and hmtR2 626 (5′- TTTATGGGGTGATGTGAGCC-3′). PCR reactions were conducted using the following cycling parameters: initial denaturation at 94 °C for 2 min; 10 cycles of 94 °C for 15 s, 65 °C for 30 s, and 68 °C for 5 min; 25 cycles of 94 °C for 15 s, 65 °C for 30 s, and 68 °C for 5 min; and a final extension at 68 °C for 7 min. Subsequently, a library was generated using the Nextera DNA Flex Library Prep Kit (Illumina) following the manufacturer's instructions. Paired-end sequencing was performed with generation of 150-bp reads on the MiSeq platform (Illumina). Bioinformatic processes, including alignment and annotation, were performed using NextGene Version 2.4.0.1 (Softgenetics, State College, PA, USA).

Furthermore, we performed the SALSA MLPA probemix P125-C1) (Lot#0719) (MRC-Holland, Amsterdam, the Netherlands) to detect the deletions using 32 Probes. We analyzed the amplification products using an ABI PRISM 3130 Genetic Analyzer (Applied Biosystems, Foster City, CA) and interpreted the results using Gene Marker 1.91 software (SoftGenetics, State College, PA).

Immunocytochemistry and EdU labeling assay

A549 cells were prepared for immunofluorescence microscopy, involving transfection, fixation, permeabilization, and antibody incubation. The cells were then mounted with DAPI-containing medium and imaged using a Leica STELLARIS 8 microscope. During EdU labeling, cells were treated with EdU, visualized with Alexa Fluor 488, and had their mitochondria stained with MitoTracker Red. Quantitative analysis involved counting EdU foci in images, normalized to cell size.

Electrophoretic mobility shift assay

For the Electrophoretic Mobility Shift Assay (EMSA) targeting SSBP1, a specific ssDNA probe was employed. Both wild-type and mutant variants of SSBP1 proteins were combined with the probes and incubated for 30 minutes. Following this incubation, samples were subjected to electrophoresis on 2% agarose gels at 100 V for 45 minutes. Following electrophoresis, the gels were visualized using UV light.

SDS-PAGE and immunoblotting

Cell lysates were mixed with sample buffer and denatured for SDS-PAGE. Proteins were then transferred to PVDF membranes, blocked, and incubated with primary and secondary antibodies. Protein bands were visualized using chemiluminescence.

Fibroblast cell culture

A skin biopsy was taken from a donor under local anesthesia and stored in Phosphate Buffered Saline (PBS). This biopsy was subsequently sectioned into 9-12 distinct segments, which were then seeded in a 12-well plate supplemented with DMEM and 20% FBS. Upon reaching confluence, the fibroblasts were

harvested for expansion.

Senescence assay

Patient-derived fibroblasts and edited cells were seeded in 6-well plates. After a 16-hour incubation period, the cells were carefully rinsed with PBS and subsequently fixed with a 1× fixative solution provided by a senescence β-galactosidase staining kit (9860, Cell Signaling Technology). The fixation process was carried out for 20 minutes at room temperature. A fresh β-galactosidase staining solution was meticulously prepared in accordance with the manufacturer's instructions. Following two PBS washes, each well was treated with 1 mL of the staining solution. Cells exhibiting β-galactosidase positivity were identified as senescent cells, and their population was quantified by analyzing a minimum of >500 cells. All images in each figure were captured using identical microscope settings and are representative of the entire cell population.

Table S1. Summary of reported *SSBP1* mutations, including this study, and its pathogenicity prediction analysis (Attach in Excel format)

Abbreviations:

HGVS: Human Genome Variation Society (https://www.hgvs.org/)

REVEL: Rare Exome Variant Ensemble Learner (https://sites.google.com/site/revelgenomics/)

SIFT: Sorting Intolerant From Tolerant (https://sift.bii.a-star.edu.sg/)

ClinPred: Prediction tool to identify disease-relevant nonsynonymous single nucleotide variants (https://sites.google.com/site/clinpred/)

KRGDB: Korean Reference Genome Database (http://coda.nih.go.kr/coda/KRGDB/index.jsp)

ACMG/AMP 2015 guideline (http://wintervar.wglab.org/)

* Analysis of ACMG/AMP guidelines for conflicting or uncertain significance variants in the Clinvar database

Table S2. Summary of reported genotypes and phenotypes associated with *SSBP1* mutations.

Reference	Family (number)	Affected patients (number)	Gene	Mutation	Zygosity	mtDNA depletion	mtDNA deletion	Clinical phenotypes
Kullar et al	1	15	*SSBP1*	NM_001256510:c.3G>A	heterozygote	O	O	Sensorineural deafness
			MT-RNR1	m.1555A>G	homoplasmy			
Jurkute et al	2	5	*SSBP1*	NM_001256510.1:c.113G>A	heterozygote	NA	NA	Optic atrophy, Foveopathy
	1	12	*SSBP1*	NM_001256510.1:c.320G>A	heterozygote	NA	NA	Optic atrophy, Foveopathy
	1	1	*SSBP1*	NM_001256510.1:c.422G>A	heterozygote	NA	NA	Optic atrophy
Piro-Mégy et al	2	29	*SSBP1*	NM_003143.3:c.113G>A	heterozygote	O	X	Optic atrophy, sometimes foveopathy
	3	3	*SSBP1*	NM_003143.3:c.320G>A	heterozygote	O	X	Optic atrophy, sometimes foveopathy
Gustafson et al	1	1	*SSBP1*	NM_003143.3:c.79G>A	heterozygote	O	O	Infantile anemia, bone marrow failure, growth failure, ptosis, ophthalmoplegia, ataxia, retinal dystrophy, Sensorineural deafness, kidney disease, metabolic strokes, multiple endocrine deficiencies
				m.8629_14068del5440				
Del Dotto et al	1	1	*SSBP1*	NM_003143.3:c.119G>T	heterozygote	O	X	optic atrophy, retinal degeneration, nephropathy, Sensorineural deafness
	1	3	*SSBP1*	NM_003143.3:c.184A>G	heterozygote	O	X	optic atrophy, retinal macular dystrophy,

								Sensorineural deafness, nephropathy
	1	2	*SSBP1*	NM_003143.3:c.320G>A	heterozygote	O	X	optic atrophy, retinal macular dystrophy, Sensorineural deafness, nephropathy
	1	1	*SSBP1*	NM_003143.3:c.331G>C	heterozygote	O	X	optic atrophy
	1	1	*SSBP1*	NM_003143.3:c.394A>G	homozygote	O	X	retinal dystrophy, Sensorineural deafness, cardiomyopathy, ataxia, nephropathy, growth retardation
Jurkute et al	1	3	*SSBP1*	NM_003143:c.151A>G	heterozygote	NA	NA	Myopia, Optic atrophy, Retinal dystrophy, Foveopathy, Attenuated retinal vessels
	1	1	*SSBP1*	NM_003143:c.113G>A	heterozygote	NA	NA	Optic atrophy, Retinal dystrophy, Attenuated retinal vessels, Myopia
	1	1	*SSBP1*	NM_003143:c.320G>A	heterozygote	NA	NA	Optic atrophy, Retinal dystrophy, Attenuated retinal vessels, Myopia
	1	1	*SSBP1*	NM_003143:c.380G>A	compound heterozygote	NA	NA	Optic atrophy, Retinal dystrophy, Attenuated retinal vessels, Early cataracts, Myopia
			SSBP1	NM_003143:c.394A>G		NA	NA	
	1	1	*SSBP1*	NM_003143:c.335G>A	heterozygote	NA	NA	Retinal dystrophy

Meunier et al	1	27	*SSBP1*	NM_003143.3:c.113G>A	heterozygote	X	X	optic atrophy, visual impairment, foveopathy
Lee et al	1	1	*POLG*	NM_002693.3_c.868C>T	heterozygote	N/A	O	sideroblastic anemia, pancytopenia, bone marrow failure, proximal renal tubular acidosis, hronic kidney disease, exocrine pancreatic insufficiency, adrenal cortical insufficiency, developmental delay
			SSBP1	NM_003143.3:c.320G>A	heterozygote			
Jun et al	1	2	*SSBP1*	NM_003143.3:c.364A>G	heterozygote	O	X	optic atrophy, diffuse retinal nerve fiber layer thinning
Chang et al	1	1	*SSBP1*	NM_003143.3:c.320G>A	heterozygote	NA	NA	Optic atrophy, color blindness
This study	1	1	*SSBP1*	NM_003143.3:c.272G>A	heterozygote	O	X	Sensorineural deafness, optic atrophy, myopathy, early cataract, macular dystrophy

Abbreviations: O, presence; X, absence; NA, not applicable.

Table S3. Oligonucleotides sequences used in this study

**Human *SSBP1* primer pair (cDNA)**<br>Forward : 5'-AAGATCCCTGAATCGTGTGC-3'<br>Reverse : 5'-TCGAGACCCCTTTTTCACAT-3'
**Human *7s DNA* primer pair (gDNA)**<br>Forward : 5'-GTGGCTTTGGAGTTGCAGTT-3'<br>Reverse 1 (7s + D-Loop) : 5'-CAGCCACCATGAATATTGTAC-3'<br>Reverse 2 (7s + D-Loop) : 5'-GAAGCAGATTTGGGTACCAC-3'
**Human *ND1* primer pair (gDNA)**<br>Forward : 5'-TACGGGCTACTACAACCCTTC-3'<br>Reverse : 5'-ATGGTAGATGTGGCGGGTTT-3'
**Human *ND5* primer pair (gDNA)**<br>Forward : 5'-CATTACTAACAACATTTCCCCCGC-3'<br>Reverse : 5'-GGCTGTGAGTTTTAGGTAGAGGG-3'
**Human *SLCO2B1* primer pair (gDNA)**<br>Forward : 5'-CCTGATGCCTAGGTTTCTTTTCTTG-3'<br>Reverse : 5'-GGTCATCTGCCTACCCTAGAAC-3'
**Human *SERP1NA1* primer pair (gDNA)**<br>Forward : 5'-CAGTGAATAAATGAGGCGTACATCC-3'<br>Reverse : 5'-GACTGTTTCTCATGCCTCTGGAAAG-3'
**Human *SSBP1* primer pair-probe set (gDNA)**<br>Forward : 5'-AGGTGATGTCAGTCAAAAGA-3'<br>Probe For WT: HEX- ATCAGTATTCCGGCCAGGCCTCA -BHQ1<br>Probe For MUT: FAM- ATCAGTATTCCAGCCAGGCCTCA -BHQ1<br>Reverse : 5'-CCATGAGAACACTTCTTATCG-3'
**Long-range PCR 1.59 kb amplicon primer pair (gDNA)**

D-loop Forward : 5'-TGGCCACAGCACTTAAACACATCTC-3'<br><br>D-loop Reverse : 5'-GGAGTTGCAGTTGATGTGTG-3'
**Long-range PCR 11.2 kb amplicon primer pair (gDNA)**<br><br>ND2 Forward : 5'-TTGCCCAAATGGGCCATTAT-3'<br><br>D-loop Reverse : 5'-GGAGTTGCAGTTGATGTGTG-3'
**Long-range PCR 9.728 kb amplicon primer pair (gDNA)**<br><br>COX1 Forward : 5'-GACCGTTGACTATTCTCTAC-3'<br><br>CYTB Reverse : 5'-GGATGGATAGTAATAGGGCA-3'
**Human control siRNA for sense**<br><br>5'-CCUCGUGCCGUUCCAUCAGGUAGUU-3'
**Human control siRNA for anti-sense**<br><br>5'-CUACCUGAUGGAACGGCACGAGGUU-3'
**Human *SSBP1* siRNA #1 for sense**<br><br>5’- CAACAAUCAUAGCUGAUAAUAUU-3’
**Human *SSBP1* siRNA #1 for anti-sense**<br><br>5’-UAUUAUCAGCUAUGAUUGUUGUU-3’
**Human *SSBP1* siRNA #2 for sense**<br><br>5’-UAAUACAGGUCUUCGAAACAUUU-3’
**Human *SSBP1* siRNA #2 for anti-sense**<br><br>5’-AUGUUUCGAAGACCUGUAUUAUU-3’
**Electrophoretic mobility shift assay probe**<br><br>5'-GGACTATTTATTCAATATATTTAAGAACTAATTCCAGCTGAGCGCCGG-3′

Table S4. Antibodies used in this study

Name	Company	cat.no
anti-DNA mouse monoclonal	PROGEN	AC-30-10
MitoTracker™ Red CMXRos	Invitrogen	M7512
Anti-SSBP1 antibody	St John's Laboratory	STJ95791
SSBP1 Polyclonal antibody	proteintech	12212-1-AP
Monoclonal Anti-Flag M2 antibody produced in mouse	Sigma	F3165-2MG
Total OXPHOS Human WB antibody cocktail	abcam	ab110411
Anti-beta Actin antibody (AC-15)	abcam	ab6276
Anti-c-Myc (phospho S62) antibody	abcam	ab51156
Goat anti-Mouse IgG(H+L)-HRP	GenDEPOT	SA001-500
Goat anti-Rabbit IgG(H+L)-HRP	GenDEPOT	SA002-500
Goat Anti-Rabbit IgG H&L (Alexa Fluor 488)	abcam	ab150077
Goat Anti-Rabbit IgG H&L (Alexa Fluor 555)	abcam	ab150078
Goat Anti-Mouse IgG H&L (Alexa Fluor 488)	abcam	ab150113
Goat Anti-Mouse IgG H&L (Alexa Fluor 647)	abcam	ab150115

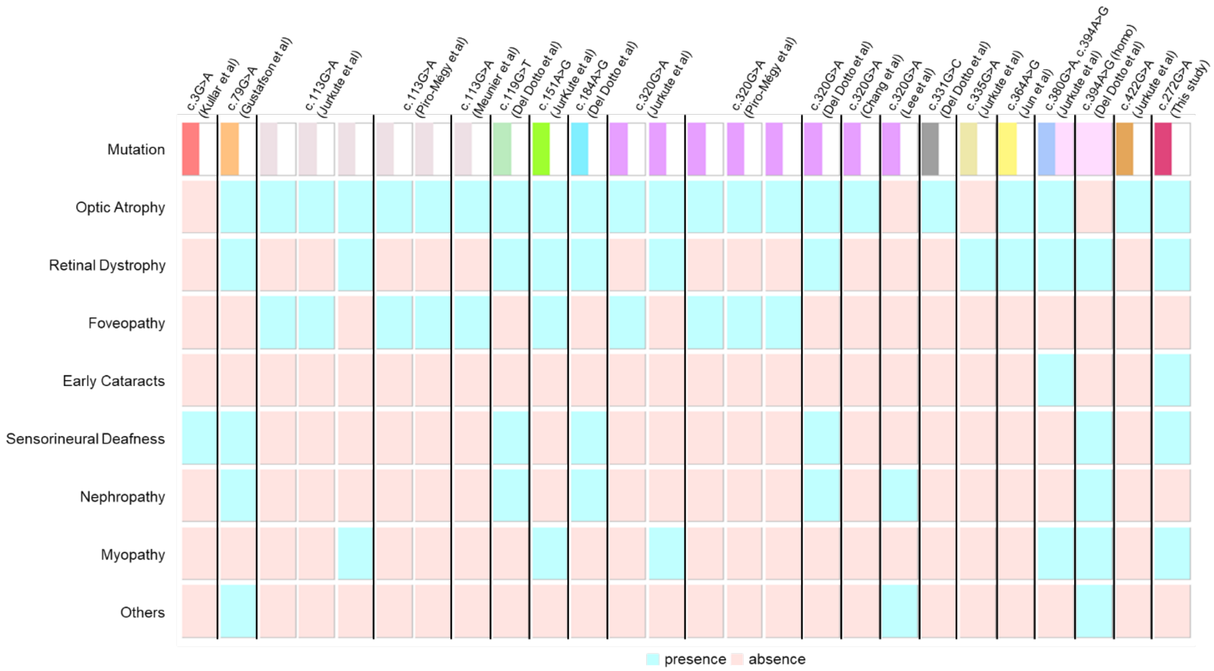

Figure S1. Representation of genotypes and clinical phenotypes of patients with *SSBP1* mutations

Our rare-grid plot comprises 14 mutations from 26 distinct families. Each column in this grid captures the genotype and clinical phenotype of an individual proband with a genetic completion. We conducted review of the clinical phenotypes documented in the literature, including optic atrophy, retinal dystrophy, foveopathy, early cataract, sensorineural deafness, nephropathy, myopathy, and other related conditions. In this illustration, blue squares indicate the presence of a particular phenotype, while red squares denote its absence. “Others” described in the rare-grid plot (y-axis) encompass anemia, bone marrow failure, ptosis, ophthalmoplegia, ataxia, metabolic strokes, multiple endocrine deficiencies, cardiomyopathy, pancytopenia, exocrine pancreatic insufficiency, adrenal cortical insufficiency, and developmental delay.

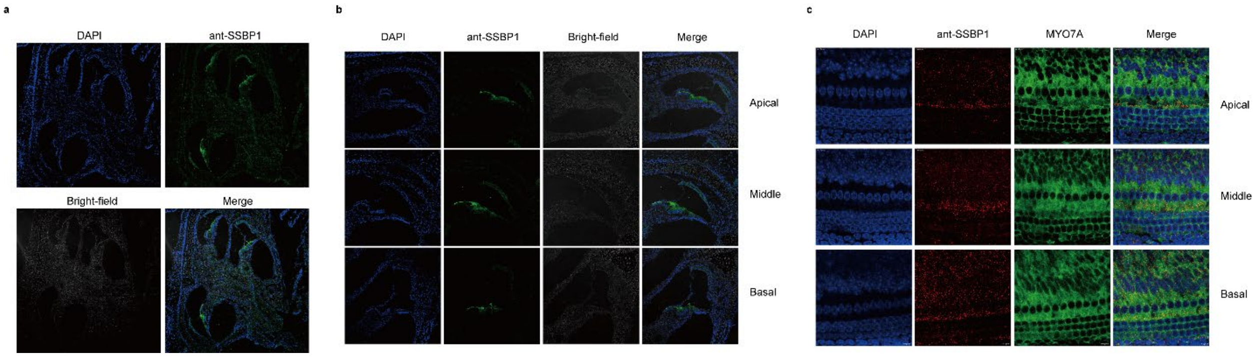

Figure S2. Immunohistochemical analysis of SSBP1 expression in the inner ear of P5 mice

(a-b) Cochlear Overview: SSBP1 expression is ubiquitously observed throughout various structures in the cochlea. This includes the outer and inner hair cells of the organ of Corti, stria vascularis, spiral ligament, and spiral ganglion cells. The SSBP1 expression is notably expressed in hair cells of the organ of Corti and stria vascularis. (c) In the merged whole-mount image, there is a noticeable overlap between SSBP1 and Myosin VIIA, visible across the basal, middle, and apical turns. Myosin VIIA is stained by antibodies targeting cochlear hair cells. Moreover, SSBP1 is distinctly expressed in the supporting cells. The antibodies used are as follows: anti-SSBP1 (STJ95791-20, St John's Laboratory) and anti-Myo7A (sc-74516, Santa Cruz Biotechnology).

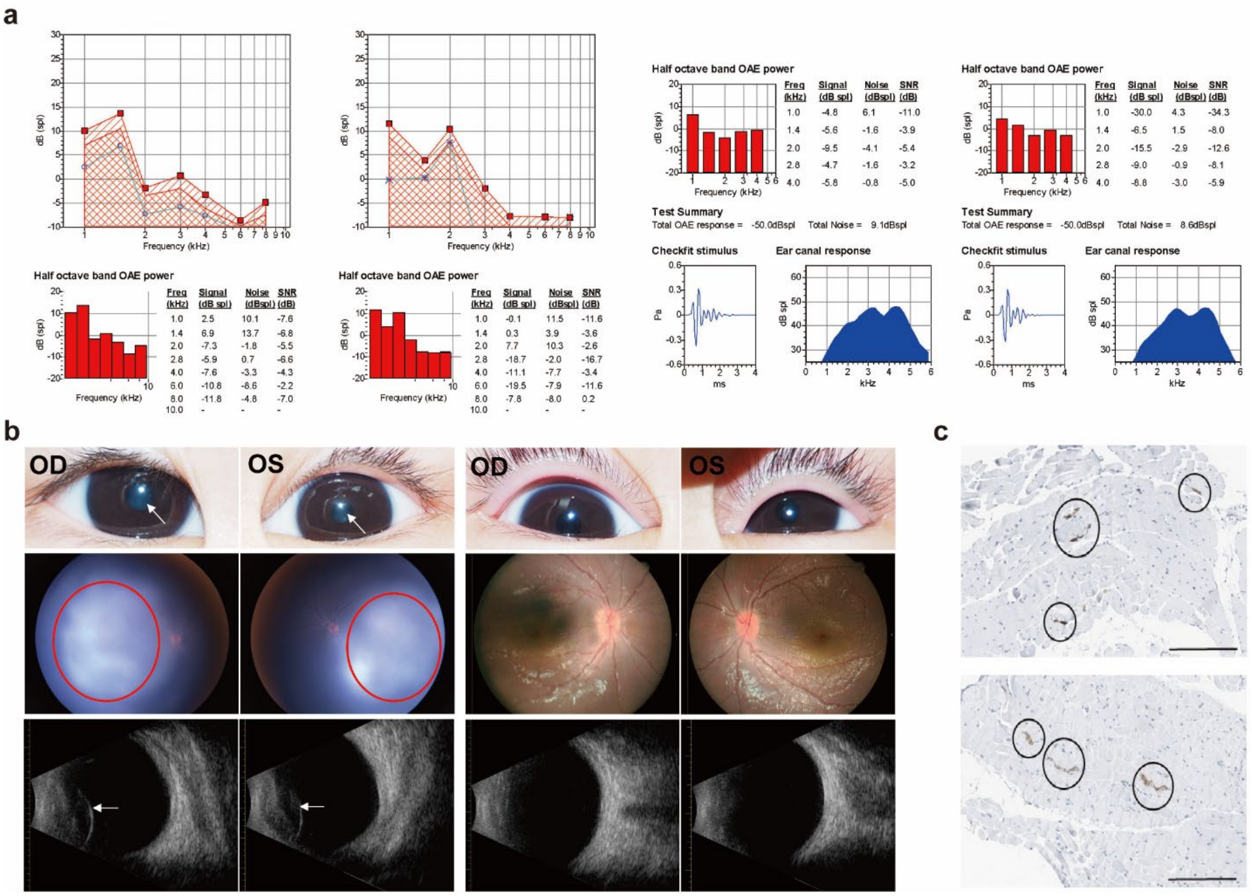

Figure S3. Clinical phenotypes observed in the proband

(a) In the Distortion Product Otoacoustic Emissions (DPOAE) test (left panel), there is no detectable otoacoustic emission, and the signal (blue) to noise (red) ratio (SNR ratio) shows significantly low dB levels across all tested frequencies. In the Transient Evoked Otoacoustic Emissions (TEOAE) test (right panel), a subnormal SNR ratio is observed across all tested frequencies, and the reproducibility is very low. These results suggest its functional loss of outer hair cells in the cochlea. (b) Ocular images from the proband before and after cataract surgery. Left panel: At the initial visit in the department of ophthalmology, the anterior segment photograph (upper) showed remarkable lens opacity (arrow) in both the right eye (OD) and the left eye (OS). An invisible macular area (red circular dashed area) with a generalized blurry fundus due to lens opacity was observed in both eyes on fundus photography (middle), and predominant lens opacity and swelling (arrow) with mild vitreous opacity near the posterior capsule of the lens on

ultrasonography (bottom) were detected in both eyes. Right panel: After four to five months cataract surgery (lensectomy and anterior vitrectomy) in both eyes at one-month intervals, the anterior photograph (upper) shows a clear visual axis with an aphakic state in both eyes. Fundus photography (middle) revealed optic disc atrophy and degeneration of the retinal pigment epithelium in the macula of both eyes. Ultrasonography (bottom) revealed mild vitreous opacity, but no other complications, such as retinal traction or vitreous hemorrhage, were detected in either eye. (c) Immunohistology images of CD56 staining of left thigh muscle tissue taken from proband's muscle biopsy. Ultrathin sections revealed myofibers with mild size variation, including both degenerating and regenerating myofibers, which predominantly appear rounded. Within the subsarcolemmal area, a few myelin, glycogen particles, and fat vacuoles are discernible. A moderate degree of endomysial fibrosis is evident. The presence of CD56-positive cells (black circles), indicating degenerating myofibers (16/10 HPF), was also noted. These findings suggest a mild myopathic change, consistent with mitochondrial myopathy.

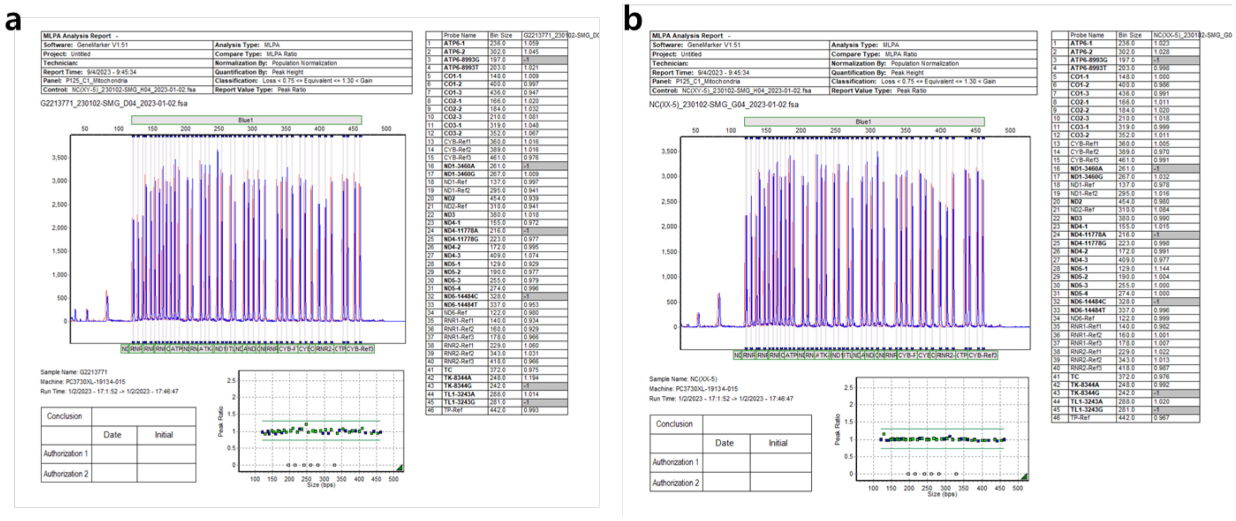

Figure S4. MLPA analysis to identify copy number variations in mitochondria genome isolated from patient's blood sample and fibroblast cells

(a) Absence of copy number variations in patient's blood sample. (b) Absence of copy number variantions in patient's fibroblast cells.

(a)

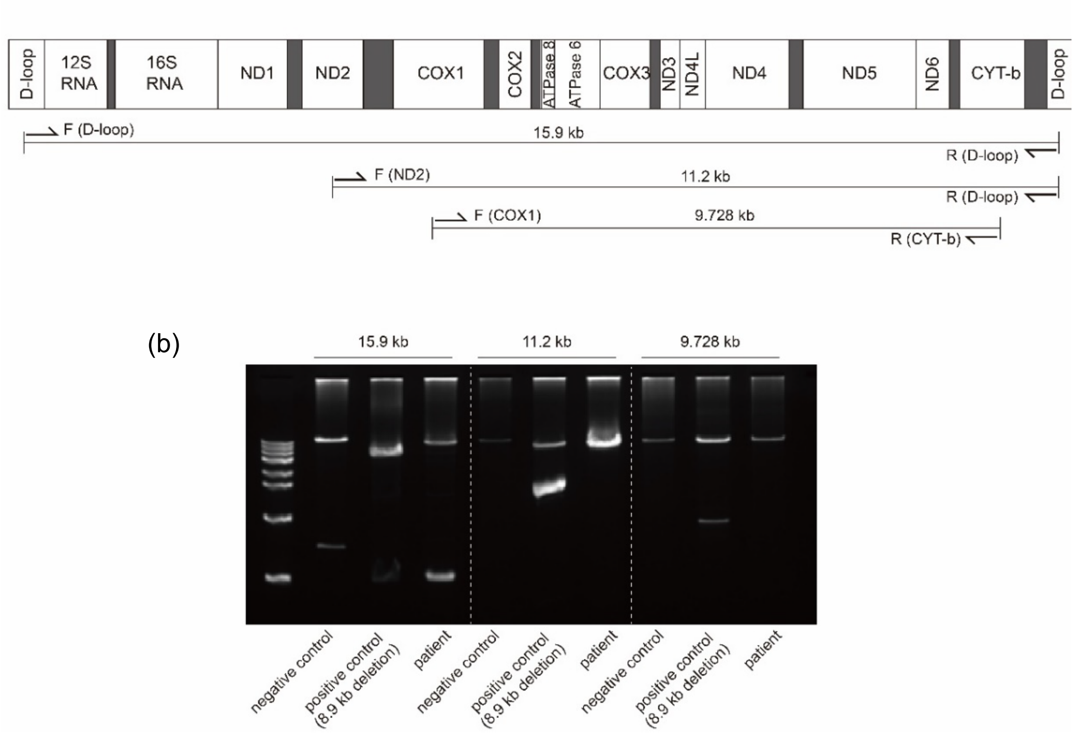

Figure S5. Long-range PCR did not show any evidence of single mtDNA deletions in the patient

(a) Location of the mtDNA products amplified with our long-range PCR primer pairs: 15.9 kb (321-16271), 12.2 kb (5250-16271), 9.728 kb (5913-15661). (b) Long-range PCR was performed on genomic DNA obtained from three sources: a negative control without mtDNA deletions, a positive control harboring an 8.9 kb mtDNA deletion, and genomic DNA isolated from the proband's skeletal muscle.

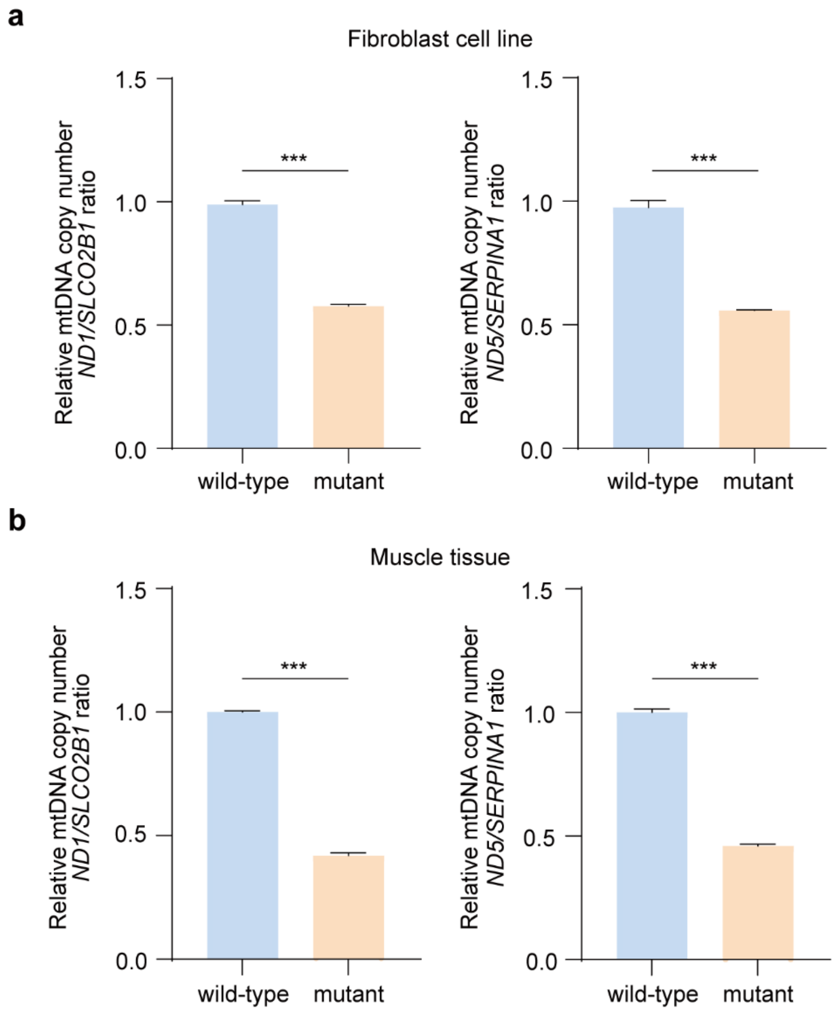

Figure S6. Long-range PCR did not show any evidence of single mtDNA deletions in the patient

(a, b) Quantification of mtDNA was performed using genomic DNA isolated from fibroblast cells (upper) and muscle tissue (lower). Relative copy numbers of mtDNA genes *ND1* and *ND5* were measured using qRT-PCR. *SLCO2B1* and *SERPINA* DNA levels were used to normalize the results; **, $p < 0.01$, ***, $p < 0.001$ (mean ± SEM, n = 3 independent experiments).

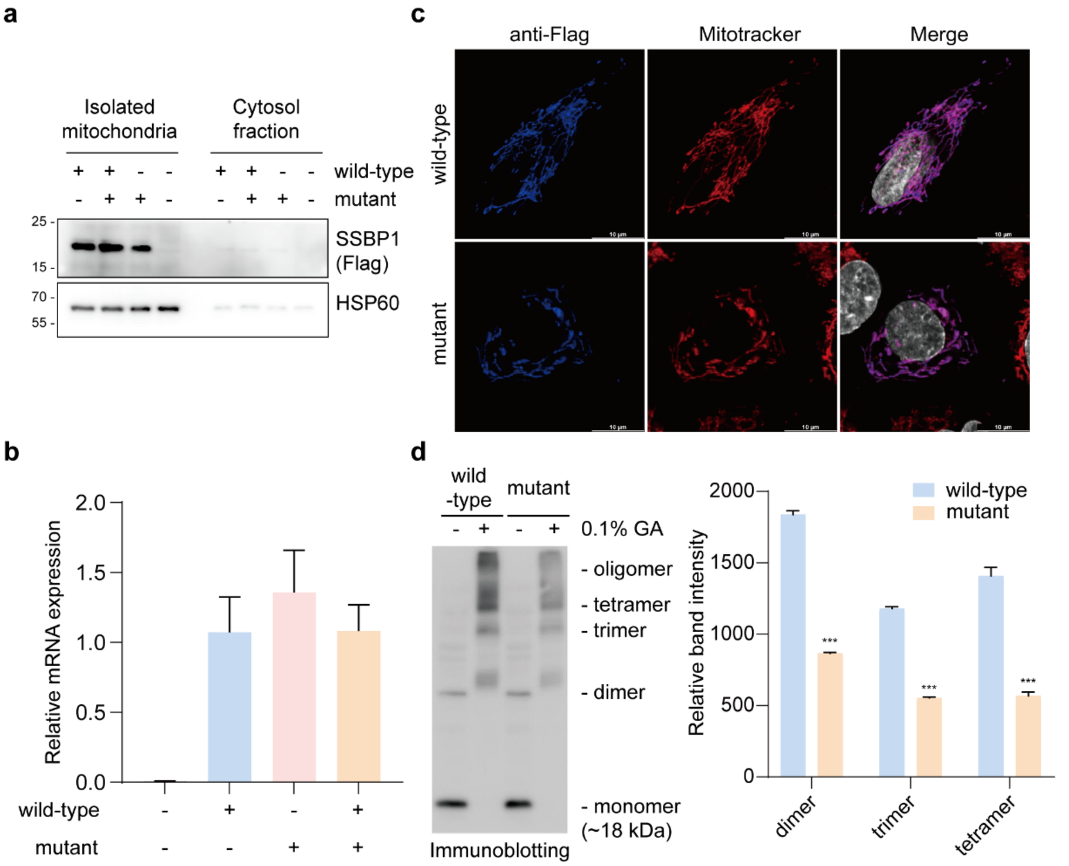

Figure S7. Comprehensive analysis of wild-type and mutant SSBP1 in cells: subcellular localization, mRNA expression and protein oligomerization

(a) Immunoblots for Flag-tagged SSBP1 and HSP60 in mitochondrial and cytosolic fractions isolated from A549 cells overexpressing wild-type and mutant *SSBP1.* (b) qRT-PCR of *SSBP1* mRNA expression in A549 cells overexpressing wild-type and mutant *SSBP1*. *GAPDH* mRNA was used to normalize the results (mean ± SEM, n = 3 independent experiments). (c) Both overexpressed wild-type and mutant SSBP1 (anti-Flag) showed colocalization with mitochondria. SSBP1 was visualized with Anti-Flag staining (blue), while mitochondria were highlighted using MitoTracker (red) and nuclei were stained with DAPI (white). (d) SSBP1 immunoblotting after oligomerization with whole-cell lysates from SSBP1 wild-type and mutant fibroblast cells. Each immunoblot band intensity was quantified using ImageJ; ***, $p < 0.001$ (means ± SEM, n = 3 independent experiments, unpaired Student's *t* test).

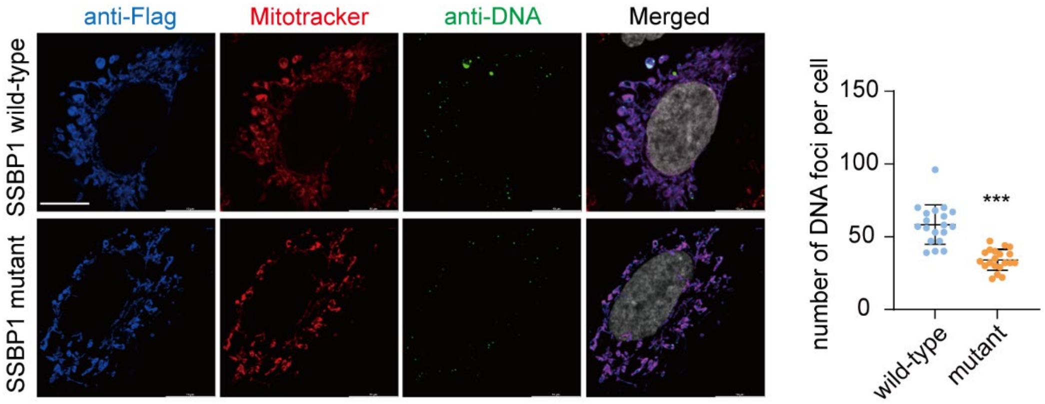

Figure S8. Mitochondrial and mtDNA staining in A549 cells expressing wild-Type or mutant SSBP1

Immunofluorescence images of Flag-tagged SSBP1 (anti-Flag, blue) in A549 cells overexpressing wild-type or mutant SSBP1. Mitochondria were detected using MitoTracker (red), and mtDNA was stained with anti-DNA (green). The number of nucleoids per cell was counted manually; ***, $p < 0.001$ (means ± SEM, n = 19-20, unpaired Student's t test). Scale bar = 10 μm.

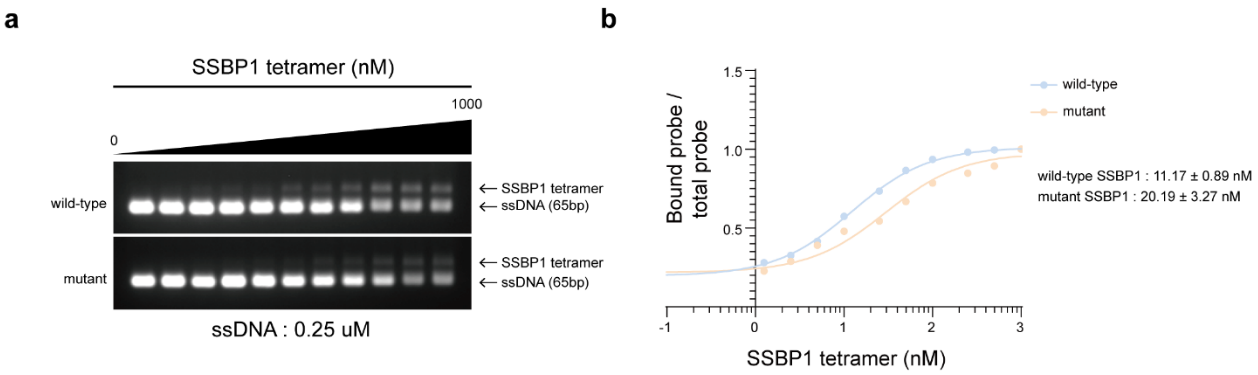

Figure S9. Electrophoresis Mobility Shift Assay (EMSA) and determination of dissociation constants for SSBP1 proteins

(a) ssDNA probe (0.25 μM) was incubated with increasing amounts (0, 0.07, 0.13, 0.25, 0.5, 1, and 2 nM) of both wild-type and mutant recombinant SSBP1 tetramers. The arrow denotes the shifted form. (b) Dissociation constants (Kd) for wild-type (11.17 ± 0.89 nM) and mutant (20.19 ± 3.27 nM) SSBP1 proteins were quantified using EMSA.

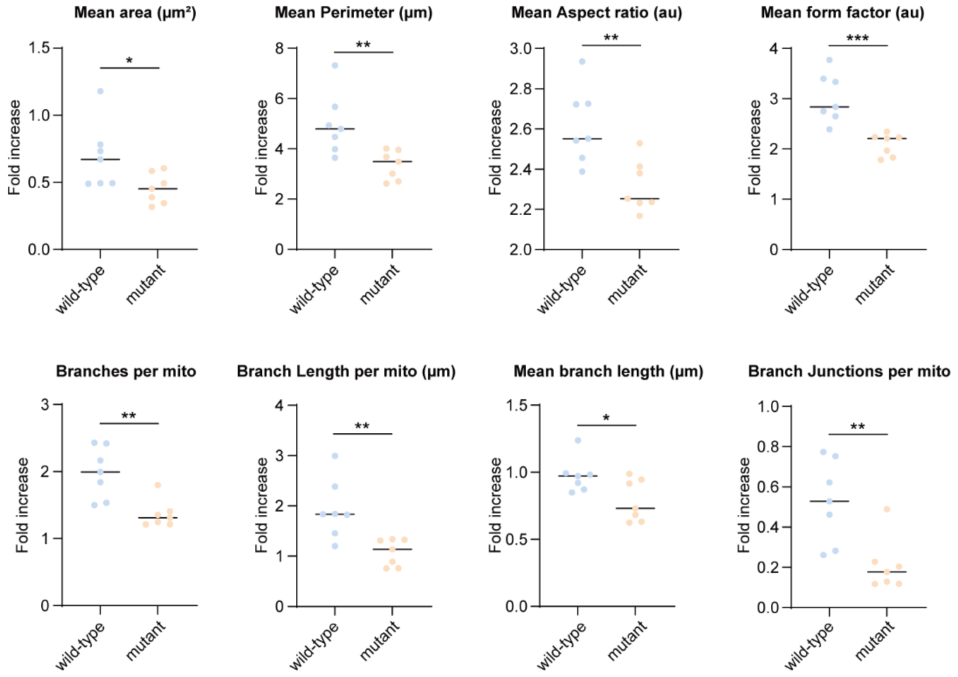

Figure S10. Impact of SSBP1 mutation on mitochondrial morphology in A549 cells

The mitochondrial networks in A549 cells overexpressing a mutant SSBP1 compared to those with wild-type SSBP1 were analyzed utilizing the Mitochondrial Network Analysis (MiNA) toolset integrated within the Fiji distribution of ImageJ. The quantitative analysis of mitochondrial parameters includes fragmentation area, perimeter, aspect ratio, form factor, branch length, number of branches, and branch junctions; *, $p < 0.1$ **, $p < 0.01$, ***, $p < 0.001$ (mean ± SEM, n = 6, unpaired Student's *t* test.).

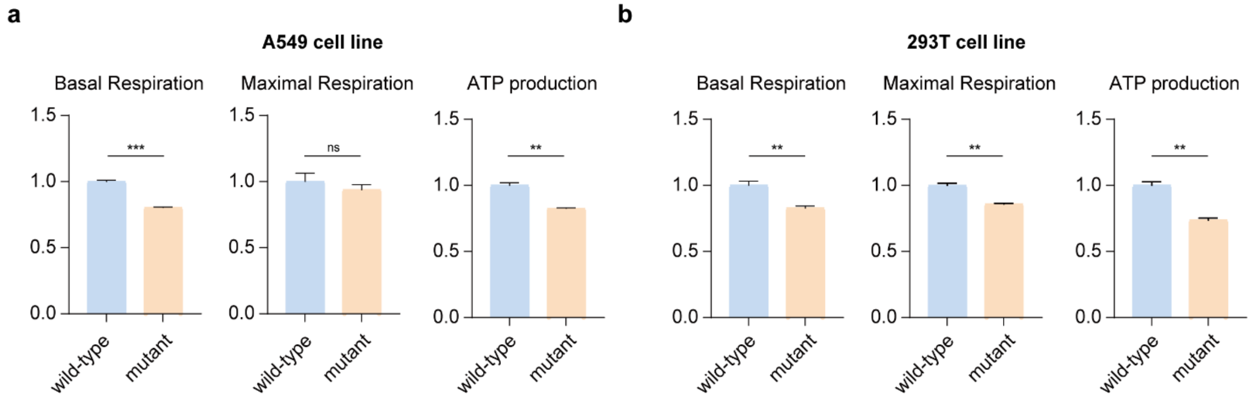

Figure S11. Changes in cellular respiration in A549 and HEK293 cells

(a, b) Alterations in basal respiration, maximal respiration, and ATP production in A549 and HEK293T cells were derived from OCR traces; ns, not significant, **, $p < 0.01$, ***, $p < 0.001$ (mean ± SEM, n = 5, unpaired Student's *t* test).

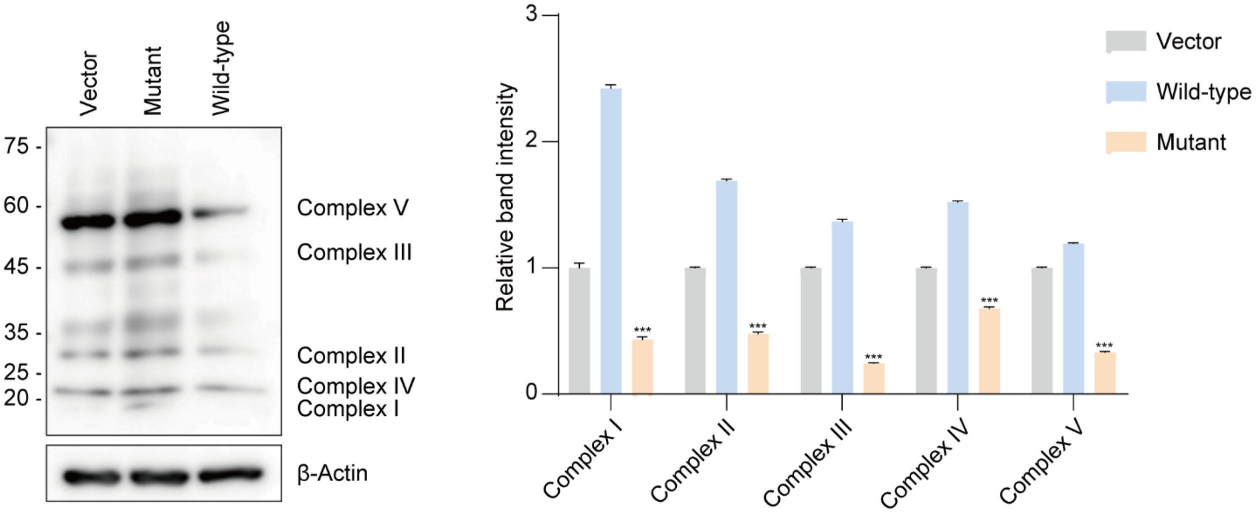

Figure S12. OXPHOS complex expression levels in A549 cells overexpressing wild-type and mutant SSBP1

Immunoblot analysis was conducted to assess the expression levels of the oxidative phosphorylation system (OXPHOS) complex and β-actin in A549 cells overexpressing either wild-type or mutant SSBP1. In cells overexpressing the wild-type SSBP1, there was an upregulation of the OXPHOS complex relative to the vector-only controls. In contrast, cells with the mutant SSBP1 exhibited a downregulation of the OXPHOS complex. The quantification of each complex was normalized to that of endogenous β-actin; ***, $p < 0.001$ (mean ± SEM, n = 3, unpaired Student's *t* test).

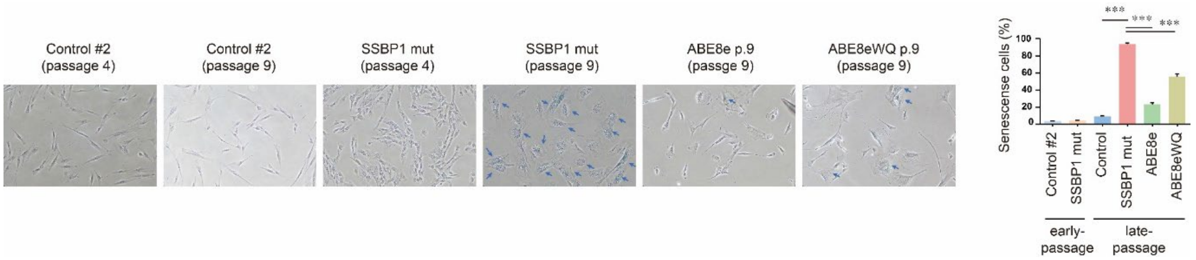

Figure S13. Cellular senescence of patient and control fibroblasts depending on the cell passages

Senescence-associated β-galactosidase (SA-β-gal) was assessed with fibroblast cell lines at both early (passage 4) and late passages (passage 9). The blue arrow points to cells that are SA-β-gal-positive. Patient-derived fibroblasts displayed an expansive cytoplasm with numerous SA-β-gal-positive cells. The relative proportion of SA-β-gal positive cells in the total cell population was quantified. Data are presented as the means ± SEM (N=10-14). ***, $p<0.005$ (one-way ANOVA followed by the Bonferroni post hoc test).

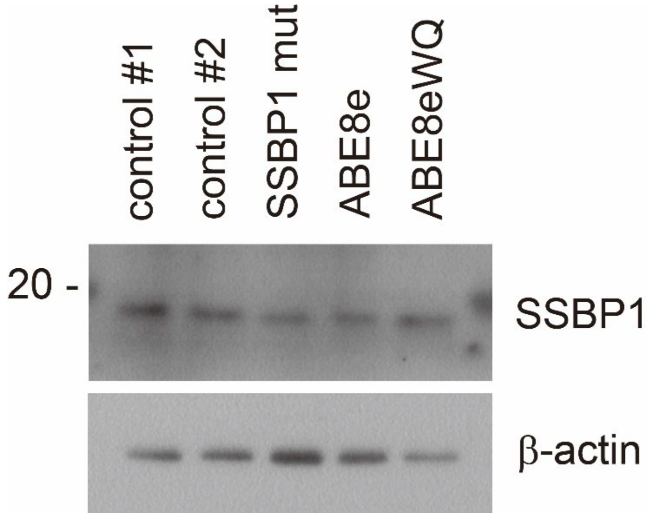

Figure S14. SSBP1 protein expression levels in whole-cell lysates derived from fibroblast cell lines

Fibroblast cell lines were lysed using RIPA lysis buffer to obtain whole-cell lysates. The extracted lysates were then separated using sodium dodecyl sulfate-polyacrylamide gel electrophoresis (SDS-PAGE). Following electrophoretic separation, the resolved proteins were transferred onto a suitable membrane and immunoblotted (IB) using the specified antibodies.

References

1. Lee, S.Y., Kim, M.Y., Han, J.H., Park, S.S., Yun, Y., Jee, S.C., Han, J.J., Lee, J.H., Seok, H., and Choi, B.Y. (2023). Ramifications of POU4F3 variants associated with autosomal dominant hearing loss in various molecular aspects. Sci Rep *13*, 12584. 10.1038/s41598-023-38272-w.
2. Lee, S., Yun, Y., Cha, J.H., Han, J.H., Lee, D.H., Song, J.J., Park, M.K., Lee, J.H., Oh, S.H., Choi, B.Y., and Lee, S.Y. (2023). Phenotypic and molecular basis of SIX1 variants linked to non-syndromic deafness and atypical branchio-otic syndrome in South Korea. Sci Rep *13*, 11776. 10.1038/s41598-023-38909-w.
3. Jo, H.D., Han, J.H., Lee, S.M., Choi, D.H., Lee, S.Y., and Choi, B.Y. (2022). Genetic Load of Alternations of Transcription Factor Genes in Non-Syndromic Deafness and the Associated Clinical Phenotypes: Experience from Two Tertiary Referral Centers. Biomedicines *10*. 10.3390/biomedicines10092125.
4. Lee, S.Y., Choi, H.B., Park, M., Choi, I.S., An, J., Kim, A., Kim, E., Kim, N., Han, J.H., Kim, M.Y., et al. (2021). Novel KCNQ4 variants in different functional domains confer genotype- and mechanism-based therapeutics in patients with nonsyndromic hearing loss. Exp Mol Med *53*, 1192-1204. 10.1038/s12276-021-00653-4.
5. Lee, S.Y., Han, J.H., Carandang, M., Kim, M.Y., Kim, B., Yi, N., Kim, J., Kim, B.J., Oh, D.Y., Koo, J.W., et al. (2020). Novel genotype-phenotype correlation of functionally characterized LMX1A variants linked to sensorineural hearing loss. Hum Mutat *41*, 1877-1883. 10.1002/humu.24095.
